# Interleukin-13 protects from atherosclerosis and modulates plaque composition by skewing the macrophage phenotype

**DOI:** 10.1002/emmm.201201374

**Published:** 2012-10-02

**Authors:** Larissa Cardilo-Reis, Sabrina Gruber, Sabine M Schreier, Maik Drechsler, Nikolina Papac-Milicevic, Christian Weber, Oswald Wagner, Herbert Stangl, Oliver Soehnlein, Christoph J Binder

**Affiliations:** 1 Department of Laboratory Medicine, Medical University of ViennaVienna, Austria; 2 Center for Molecular Medicine (CeMM) of the Austrian Academy of SciencesVienna, Austria; 3 Department of Medical Chemistry, Center for Pathobiochemistry and Genetics, Medical University of ViennaVienna, Austria; 4 Institute for Cardiovascular Prevention, Ludwig-Maximilians University MunichMunich, Germany

**Keywords:** alternatively activated macrophages (M2), atherosclerosis, cytokines, interleukin-13, oxidized LDL

## Abstract

Atherosclerotic lesions are characterized by the accumulation of oxidized LDL (OxLDL) and the infiltration of macrophages and T cells. Cytokine expression in the microenvironment of evolving lesions can profoundly contribute to plaque development. While the pro-atherogenic effect of T helper (Th) 1 cytokines, such as IFN-γ, is well established, the role of Th2 cytokines is less clear. Therefore, we characterized the role of the Th2 cytokine interleukin (IL)-13 in murine atherosclerosis. Here, we report that IL-13 administration favourably modulated the morphology of already established atherosclerotic lesions by increasing lesional collagen content and reducing vascular cell adhesion molecule-1 (VCAM-1)-dependent monocyte recruitment, resulting in decreased plaque macrophage content. This was accompanied by the induction of alternatively activated (M2) macrophages, which exhibited increased clearance of OxLDL compared to IFN-γ-activated (M1) macrophages *in vitro*. Importantly, deficiency of IL-13 results in accelerated atherosclerosis in *LDLR*^−/−^ mice without affecting plasma cholesterol levels. Thus, IL-13 protects from atherosclerosis and promotes a favourable plaque morphology, in part through the induction of alternatively activated macrophages.

## INTRODUCTION

Atherosclerosis is a chronic inflammatory disease of the artery wall, whose pathogenesis is influenced by dyslipidemia (high LDL/low high density lipoprotein, HDL) and inflammation (Hansson, 2005). Atherosclerotic lesions are characterized by the accumulation of oxidized LDL (OxLDL), the infiltration of activated macrophages and T cells, as well as cell death and fibrosis in the vessel wall of arteries (Binder et al, 2002; Hansson & Hermansson, 2011). T cells are a prominent component of the atherosclerotic plaque and exhibit signs of activation, including the expression of cytokines (Ait-Oufella et al, 2011; Hansson & Hermansson, 2011). Substantial evidence supports that T helper (Th) 1-driven responses, mainly characterized by IFN-γ production, are detrimental and correlate with progression of atherosclerosis (Buono et al, 2003, 2005; Gupta et al, 1997; Tellides et al, 2000). These pro-atherogenic responses have been shown to be dampened by the presence of specific regulatory T cells, which secrete the anti-atherogenic cytokines transforming growth factor (TGF)-β and interleukin (IL)-10 (Ait-Oufella et al, 2011). Indeed, IL-10 secreted by T cells decreases atherogenesis in mice (Mallat et al, 1999; Pinderski Oslund et al, 1999). On the other hand, the role of Th2 responses in lesion formation is more complex. For example, previous studies reported a pro-atherogenic role for IL-4 (Davenport & Tipping, 2003; King et al, 2002), while a later report found no effect (King et al, 2007). We have shown previously that the atheroprotective immunization of *LDLR*^−/−^ mice with malondialdehyde-modified LDL (MDA-LDL) induced a Th2-biased immune response that was characterized by antigen-specific production of IL-5 and IL-13 but only small amounts of IL-4 and IFN-γ. In the same study, we demonstrated the capacity of IL-5 to stimulate natural atheroprotective IgM specific for OxLDL and its ability to protect from atherosclerosis (Binder et al, 2004).

In addition to their role in providing help for B cells to secrete antibodies, another potentially important function of cytokines in atherogenesis is their capacity to modulate the activation state of macrophages. Continuous recruitment of monocyte/macrophages into lesions has been shown to be related to plaque progression (Gautier et al, 2009; Peters & Charo, 2001; Potteaux et al, 2011; Swirski et al, 2006). IFN-γ (and LPS) can promote classical activation of macrophages (M1), which is known to be pro-inflammatory and may promote atherogenesis (Khallou-Laschet et al, 2010). On the other hand, IL-4 and/or IL-13 induce alternative macrophage activation (M2), which results in potent anti-inflammatory and tissue repair capacities (Gordon & Martinez, 2010). Thus, the cytokine profile of the microenvironment can profoundly affect the activation state of macrophages and their function, *e.g.* in foam-cell formation. Recently, Khallou-Laschet et al (2010) showed that lesion-infiltrated macrophages of young *ApoE*^−/−^ mice exhibit predominantly the M2 phenotype, while M1 macrophages were dominant in more advanced lesions of aged mice and their presence correlated with lesion progression. These data suggest a potentially atheroprotective role of anti-inflammatory M2 macrophages and the cytokines involved in alternative macrophage activation. Importantly, the presence of anti-inflammatory M2 macrophages has also been documented in human carotid atherosclerotic lesions (Bouhlel et al, 2007).

IL-13 is exclusively produced by hematopoietic cells, including activated Th2 cells and the recently discovered nuocytes (Barlow & McKenzie, 2011; Oliphant et al, 2011). Because IL-4 and IL-13 share a common receptor signalling pathway (IL-4Rα/IL-13Rα1/STAT6) similar functions for both cytokines have been described (Chomarat & Banchereau, 1998; Kuperman & Schleimer, 2008). However, IL-13 also possesses functions that differ from those of IL-4 (Chiaramonte et al, 1999; Kumar et al, 2002; Liang et al, 2011; McKenzie et al, 1998b; Oriente et al, 2000). For example, IL-13 can initiate signalling via the alternative IL-13 receptor IL-13Rα2, which exclusively binds IL-13 and which has been shown to induce TGF-β1 production in macrophages via a STAT6-independent pathway, leading to collagen deposition *in vivo* (Fichtner-Feigl et al, 2006). Although it has been assumed that IL-13 affects atherosclerosis in the same way as IL-4, no studies are currently available to support this notion (Tedgui & Mallat, 2006). Therefore, we tested the role of IL-13 in atherosclerotic lesion formation. Based on the prominent functions of IL-13 in inducing fibrosis and alternative macrophage activation, we hypothesized that IL-13 might have an atheroprotective role by modulating plaque composition.

Here, we demonstrate that exogenous administration of IL-13 to cholesterol-fed *LDLR*^−/−^ mice promotes collagen formation and reduces macrophage content of existing lesions. The latter was confirmed in cholesterol-fed *ApoE*^−/−^ mice, and found to be a consequence of decreased vascular cell adhesion molecule-1 (VCAM-1)-dependent monocyte recruitment. In addition, IL-13 administration induces a switch of the lesional macrophage phenotype *in vivo* towards alternative activation. Macrophages, which were alternatively activated with IL-13 *in vitro*, have an increased capacity of clearing OxLDL by promoting its uptake as well as cholesterol efflux without increasing net foam-cell formation. Moreover, we show that *LDLR*^−/−^ mice that were reconstituted with bone marrow of *IL-13*-deficient mice develop accelerated atherosclerosis.

## RESULTS

### IL-13 administration modulates established atherosclerosis

**Figure 1:**
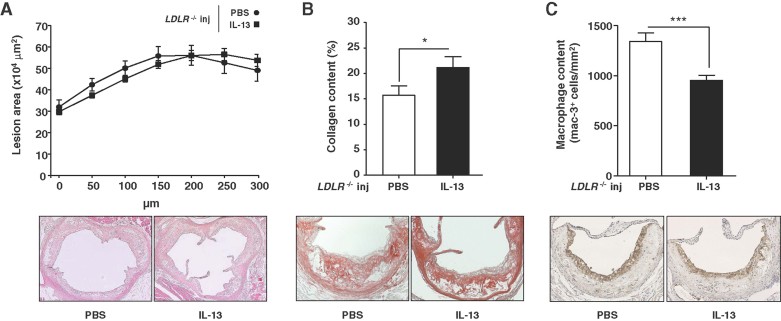
IL-13 administration alters plaque morphology of established atherosclerotic lesions *LDLR*^−/−^ mice were fed an atherogenic diet for 16 weeks and received biweekly intraperitoneal injections with PBS (*n* = 11) or IL-13 (*n* = 13) during the last 5 weeks. Equal extent of atherosclerotic lesion size in cross-sections of the aortic origin in injected *LDLR*^−/−^ mice. Values represent µm^2^/section throughout the entire aortic origin (300 µm). Original magnification 50×.Increased collagen content in lesions of *LDLR*^−/−^ mice injected with IL-13. Sections were stained with Sirius Red for the presence of collagen, and values represent the percentages of Sirius Red^+^ area/total lesion area. *p = 0.035. Original magnification 100×.Decreased macrophage content in lesions of *LDLR*^−/−^ mice injected with IL-13. Sections were stained with the macrophage-specific anti-mac-3 antibody and values represent the numbers of mac-3^+^ cells/mm^2^ of total lesion area. ***p = 0.0007. Original magnification 100×. All data are mean ± SEM values of all mice of each group. Images show representative examples of the respective stainings.

To study the capacity of IL-13 to influence existing atherosclerosis, we performed an intervention study in which IL-13 was administered exogenously to mice with established atherosclerotic lesions. *LDLR*^−/−^ mice were fed an atherogenic diet for 16 weeks and received biweekly intraperitoneal injections of IL-13 or phosphate-buffered saline (PBS) during the last 5 weeks of diet. Based on our observation that atherosclerotic *LDLR*^−/−^ mice have IL-13 serum levels of approximately 2.5 ng/ml, we decided to administer 50 ng of IL-13 per *LDLR*^−/−^ mouse twice per week, which corresponds to only threefold higher amounts of systemic IL-13 per day. At time of sacrifice, mice were not different with respect to body weight, total plasma cholesterol (TC), triglycerides (TG), or levels of total IgG1 or IgG2c antibodies (Table 1). Furthermore, there were no differences in the frequencies of splenic T or B cells (Table 1). Stimulated splenocytes from atherosclerotic *LDLR*^−/−^ mice treated with PBS or IL-13 produced similar amounts of Th2 (IL-4, IL-5, IL-10 and IL-13) and Th1 (IFN-γ) cytokines, indicating that IL-13 administration at this dose did not alter the overall Th1/Th2 balance (Fig S1 of Supporting Information). In addition, no induction of liver or lung fibrosis due to the continuous IL-13 injection was observed (unpublished observations). As expected with the relatively short time of administration only during the last 5 weeks of a 16-week feeding period, there were no differences in the extent of atherosclerosis in the cross-sectional analyses of the aortic origin or in the entire aorta by *en face* analyses between atherosclerotic *LDLR*^−/−^ mice treated with PBS or IL-13, respectively (Fig 1A and Table 1).

**Table 1:** Overview of experimental data of the IL-13 administration study

	*LDLR*^−/−^ inj → PBS (*n* = 11)	*LDLR*^−/−^ inj → IL-13 (*n* = 13)
Atherosclerosis		
Aortic origin (10^4^ µm^2^/section)	48.16 ± 3.56	47.15 ± 1.22
*En face* (% of aorta)	6.63 ± 0.94	6.22 ± 0.66
Metabolic parameters		
Weight (g)	23.6 ± 0.7	22.5 ± 0.5
TC (mg/dl)	1493 ± 49	1362 ± 93
TG (mg/dl)	863 ± 85	777 ± 81
Serum antibody titers		
Total IgM (mg/ml)	0.97 ± 0.11	0.85 ± 0.11
Total IgG1 (mg/ml)	1.11 ± 0.10	0.97 ± 0.11
Total IgG2c (mg/ml)	1.84 ± 0.16	1.58 ± 0.16
Serum chemokines		
CCL2/MCP-1 (pg/ml)	59.1 ± 8.3	69.3 ± 8.1
CXCL1/KC (pg/ml)	1709 ± 247	1535 ± 181
Characterization of PEC a		
Total peritoneal cells (×10^6^)	3.38 ± 0.52	2.22 ± 0.29
CD5^+^ T cells (% of total)	20.40 ± 3.74	15.25 ± 2.71
CD11b^+^ Mac cells (% of total)	13.96 ± 2.16	18.78 ± 2.32
CD19^+^ B cells (% of total)	53.25 ± 3.43	51.84 ± 3.29
CD11b^+^CD5^+^ B1a cells (% of B cells)	27.24 ± 2.71	24.56 ± 3.29
CD11b^+^CD5^−^ B1b cells (% of B cells)	16.95 ± 1.35	19.37 ± 1.47
CD19^+^CD23^+^ B2 cells (% of B cells)	28.43 ± 3.39	32.69 ± 3.68
Characterization of PBC b		
Total white blood cells/ml (×10^6^)	1.40 ± 0.18	1.59 ± 0.25
CD11b^+^ Ly6C^lo^ monocytes (% of total)	4.11 ± 0.34	4.96 ± 0.43
CD11b^+^ Ly6C^hl^ monocytes (% of total)	2.59 ± 0.36	2.15 ± 0.36
CD11b^+^ Ly6G^+^ neutrophils (% of total)	3.97 ± 0.46	4.51 ± 0.65
Characterization of splenocytes c		
Total spleen cells/ml (×10^6^)	67.3 ± 11.3	62.3 ± 9.4
CD43^+^ T cells/ml (% of total)	24.83 ± 2.26	23.39 ± 1.42
B220^+^ B cells/ml (% of total)	45.35 ± 3.32	46.67 ± 1.92
CD43^+^IgM^+^ B1 cells/ml (% of B cells)	16.29 ± 1.27	17.16 ± 1.22
CD43^−^IgM^+^ B2 cells/ml (% of B cells)	81.76 ± 1.21	80.97 ± 1.19

Atherosclerosis in the aortic origin was analyzed by cross-sections through the aortic origin and values represent the average µm^2^/section. *En face* measurements are given in percent lesion area of the entire aorta. TC, total serum cholesterol; TG, serum triglycerides; PEC, peritoneal exudate cells; PBC, peripheral blood cells. Data are shown as mean ± SEM.

a PEC cellular populations as measured by flow cytometry.

b PBC cellular populations as measured by flow cytometry.

c Spleen cellular populations were analyzed by flow cytometry.

As plaque size was similar between the two groups, we were able to directly compare lesion composition. Morphological analyses of the cross-sectional lesions showed no significant differences in the necrotic core area, suggesting that both groups of mice had the same stage of lesion development (Fig S2A of Supporting Information). In contrast, the collagen content was significantly higher in atherosclerotic *LDLR*^−/−^ mice treated with IL-13 compared to PBS-treated mice (Fig 1B), while the smooth-muscle cell content remained unchanged between the groups (Fig S2B of Supporting Information). These results suggest that IL-13 injections stimulated the production of collagen by either macrophages or smooth-muscle cells rather than the proliferation of these cells, consistent with the pro-fibrotic function of IL-13. In addition, the numbers of T cells were not different between the two groups (Fig S2C of Supporting Information). Remarkably, immunohistological analyses of mac-3^+^ macrophages uncovered a significant reduction in lesional macrophages in the IL-13-treated mice (Fig 1C). These differences in macrophage numbers were not due to changes in circulating blood cell counts, as total numbers of white blood cells, monocytes (both classical Ly6C^hi^ and nonclassical Ly6C^lo^) or neutrophils in the peripheral blood of mice were similar (Table 1). Moreover, the plasma levels of CCL2/MCP-1 and CXCL1/KC, the two major chemokines involved in monocyte recruitment, were not reduced in IL-13-injected mice (Table 1).

Taken together, our data showed that IL-13 modulates established atherosclerotic lesions by inducing a more stable plaque composition with higher collagen content and fewer macrophages.

### IL-13 administration reduces macrophage content in lesions of atherosclerotic *ApoE*^−/−^ mice, independent of macrophage egress

**Figure 2:**
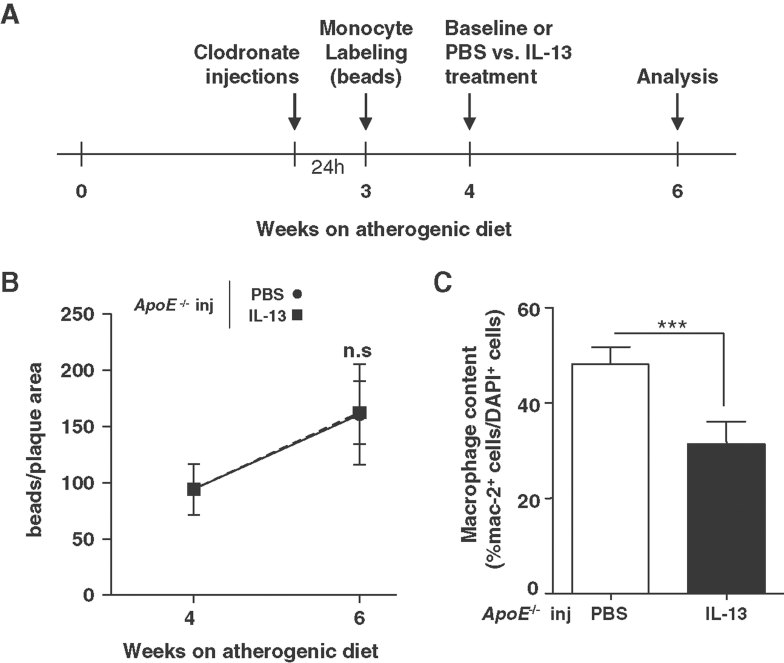
IL-13 administration has no effect on macrophage egress from established atherosclerotic lesions *ApoE*^−/−^ mice were fed an atherogenic diet for 4 weeks (baseline) or 6 weeks (PBS and IL-13, respectively). After 3 weeks, circulating monocytes of all mice were labelled with fluorescent latex beads following clodronate-liposome depletion 24 h before. One week later, one group of mice was sacrificed for baseline (*n* = 7) measurements of bead and macrophage content and the remaining mice received biweekly intraperitoneal injections with either PBS (*n* = 7) or IL-13 (*n* = 8) until sacrifice. Diagram of experimental design.Similar content of fluorescent beads per plaque area in the aortic root of PBS and IL-13 administration, respectively, compared to baseline.Decreased macrophage content in lesions of *ApoE*^−/−^ mice injected with IL-13. Sections were stained with the macrophage-specific anti-mac-2 antibody and values represent the percentages of mac-2^+^ cells/total DAPI^+^ cells. ***p = 0.0001. All data are shown as mean ± SEM values of all mice of each group.

The observed morphological changes of atherosclerotic lesions following IL-13 administration suggested the possibility of macrophage egression. Because CCR7 has been implicated as a key chemokine receptor in this, we first evaluated CCR7 expression in lesions of the cholesterol-fed *LDLR*^−/−^ mice that were treated with either IL-13 or PBS, respectively. There was no significant difference in the percentage of CCR7^+^ lesion area between the two groups, suggesting no major contribution of CCR7-mediated emigration (Fig S3 of Supporting Information). We then directly evaluated the ability of IL-13 to induce macrophage egress from existing lesions using a method that is based on the tracking of fluorescent bead-labelled monocyte/macrophages in *ApoE*^−/−^ mice (Potteaux et al, 2011). To achieve comparable lesion development in these mice, they were fed an atherogenic diet for a total of 6 weeks and received PBS or IL-13 during the last 2 weeks of diet. Following depletion of monocyte/macrophages by clodronate-liposome injection 20 days after initiation of the atherogenic diet, circulating monocytes were labelled with latex-beads to monitor lesional macrophage migration. At day 28, one-third of mice were sacrificed to obtain a baseline number of beads per plaque area in the aortic root. The other mice were divided into two groups and received biweekly injections of either IL-13 or PBS, respectively, for the remaining 2 weeks (Fig 2A). At time of sacrifice, cross-sections of the aortic origin were analyzed for the presence of fluorescent beads as well as mac-2^+^ cells. The numbers of beads per plaque area at week 6 were not different between PBS- and IL-13-injected animals and comparable to the numbers at baseline (week 4), indicating that IL-13 had no effect on macrophage egression (Fig 2B). Nevertheless, macrophage content of the same lesions was significantly lower in IL-13-injected mice compared to PBS-injected mice, thereby confirming our initial observation in a different atherosclerosis-prone mouse strain (Fig 2C).

Thus, IL-13 limits lesional macrophage content by a mechanism other than macrophage emigration.

### IL-13 administration results in decreased VCAM-1 expression and monocyte adhesion in lesions of atherosclerotic *ApoE*^−/−^ mice *in vivo*

**Figure 3:**
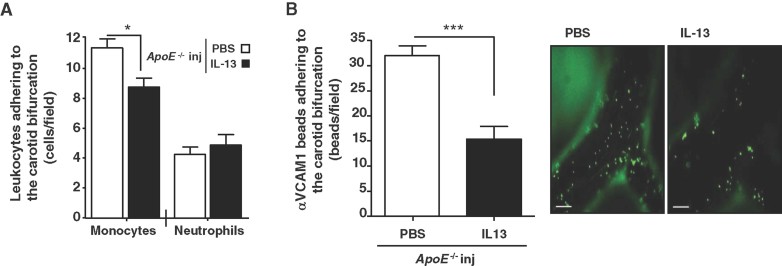
IL-13 administration reduces VCAM-1-dependent monocyte recruitment in established atherosclerotic lesions *Cx3cr1*^gfp/wt^ *ApoE*^−/−^ mice were fed an atherogenic diet for 6 weeks and received biweekly intraperitoneal injections with PBS (*n* = 8) or IL-13 (*n* = 8) during the last 2 weeks. At the time of sacrifice, PE-conjugated anti-GR1 antibodies were injected intravenously and the number of monocytes (GFP^+^ cells) and neutrophils (PE^+^ cells) adhering to the carotid bifurcation was assessed by intravital microscopy. Values represent the numbers of cells/optical field. *p = 0.0185, Mann–Whitney test.*ApoE*^−/−^ mice were fed an atherogenic diet for 6 weeks and received biweekly intraperitoneal injections with PBS (*n* = 8) or IL-13 (*n* = 8) during the last 2 weeks. At time of sacrifice, fluorescent anti-VCAM-1 beads were injected intravenously and the number of beads adhering to the carotid bifurcation was assessed by intravital microscopy. Values represent the numbers of beads/optical field. ***p = 0.0001. Representative microscopy images are shown. Bar: 100 µm. All data are shown as mean ± SEM values of all mice of each group.

To explain the decreased numbers of macrophages in atherosclerotic lesions despite unchanged peripheral blood monocyte counts, similar plasma levels of important chemokines, and no effect on macrophage egression, we hypothesized that IL-13 reduced the recruitment and adhesion of monocytes to the atherosclerotic wall. To elucidate this directly, we assessed the adhesion of different leukocyte populations by intravital microscopy of carotid arteries of Cx_3_cr1^gfp/wt^ mice that were available on an *ApoE*^−/−^ background. Following injection of a fluorescent antibody to GR1 (Ly6C/G), this animal model enabled us to differentiate, at the same time, between the adhesion of two subtypes of circulating monocytes (nonclassical Gr1^−^ Cx_3_cr1^hiGFP^ and classical Gr1^+^ Cx_3_cr1^loGFP^) and neutrophils (Gr1^+^gfp^−^). Indeed, the adhesion of circulating monocytes to carotid arteries was significantly decreased in atherosclerotic mice injected with IL-13 compared to PBS-treated mice (Fig 3A), whereas adhesion of circulating neutrophils was similar (Fig 3A). In addition, the adhesion of the two monocyte subsets was equally reduced (unpublished observation), suggesting that IL-13 administration reduces monocyte recruitment through a common pathway. Thus, we hypothesized that IL-13-induced changes may result in decreased endothelial cell activation. To test this, we performed intravital microscopy of carotid arteries of *ApoE*^−/−^ mice that were fed an atherogenic diet for a total of 6 weeks and received PBS or IL-13 during the last 2 weeks of diet, followed by the injection of fluorescently labelled beads coupled with anti-VCAM-1 or anti-CCL2 antibodies to analyze the carotid endothelial activation state in these mice. VCAM-1 expression was significantly decreased in IL-13-treated mice (Fig 3B), whereas the endothelial presentation of CCL2 was similar in both groups (Fig S4 of Supporting Information).

These data strongly suggest that the decrease in lesional macrophage content observed in IL-13-treated mice is caused by a reduction in the VCAM-1-dependent monocyte recruitment.

### IL-13 administration skews macrophage phenotype towards alternatively activated macrophages (M2) *in vivo*

**Figure 4:**
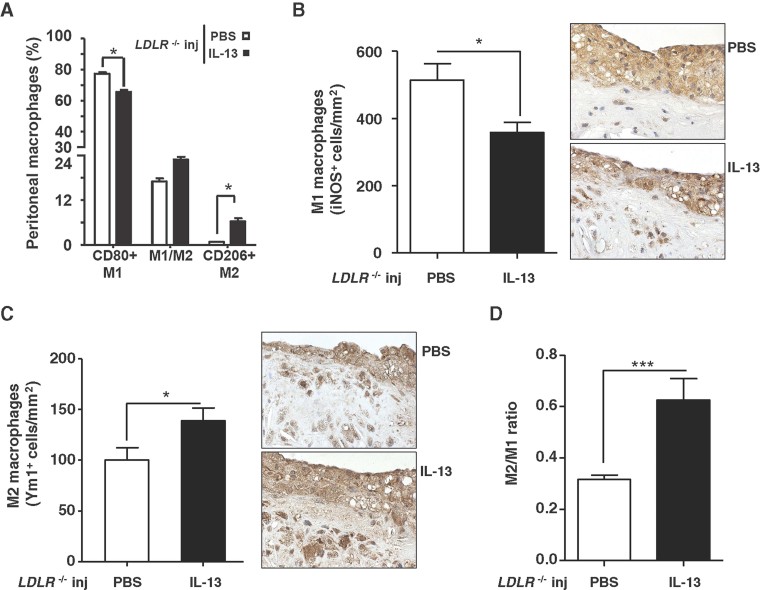
IL-13 administration skews macrophage phenotype towards alternatively activated (M2) macrophages *in vivo* *LDLR*^−/−^ mice were fed an atherogenic diet for 16 weeks and received biweekly intraperitoneal injections with PBS (*n* = 11) or IL-13 (*n* = 13) during the last 5 weeks. Increased frequencies of M2 macrophages and decreased frequencies of M1 macrophages in the peritoneal cavities of *LDLR*^−/−^ mice injected with IL-13. Peritoneal cells were stained with antibodies against CD80 (M1) and CD206 (M2) and identified by flow cytometry. CD80/CD206 double-positive cells were classified as M1/M2 macrophages. Values represent the percentages of individual macrophage subtypes/total macrophages. *p = 0.04.Decreased numbers of M1 macrophages in lesions of *LDLR*^−/−^ mice injected with IL-13. Sections were stained with an antibody against iNOS, which is specifically expressed by M1 macrophages and values represent the numbers of iNOS^+^ cells/mm^2^ of total lesion area. *p = 0.012.Increased numbers of M2 macrophages in lesions of *LDLR*^−/−^ mice injected with IL-13. Sections were stained with an antibody against Ym-1, which is specifically expressed by M2 macrophages. Values represent the numbers of Ym-1^+^ cells/mm^2^ of total lesion area. *p = 0.024.Increased ratio of M2:M1 macrophages in lesions of *LDLR*^−/−^ mice injected with IL-13. ***p = 0.0001. All data are shown as mean ± SEM values of all mice of each group. Images show representative examples of the respective stainings. Original magnification: 400×.

Because IL-13 is known to induce alternative macrophage activation, we investigated whether IL-13 administration had the capacity to generate M2 macrophages *in vivo*. Indeed, we observed a shift within the macrophage population towards more CD206^+^ macrophages (M2) and less CD80^+^ macrophages (M1) in the peritoneal cavities of IL-13-treated mice as assessed by flow cytometry (Fig 4A) and further confirmed by quantitative polymerase chain reaction (PCR), demonstrating a significant up-regulation of common M2-related genes, including arginase-1 (Arg-1), chitinase 3-like 3 protein (Chi3l3/Ym-1) as well as CCL9, and a concomitant down-regulation of M1-related genes such as inducible nitric oxide synthase (iNOS), CD86 and CXCL10 (Fig S5A of Supporting Information). The total numbers of peritoneal macrophages, T and B cells (including B1 and B2 cells) were not different between the two groups (Table 1). Importantly, in analogy to the activation state of peritoneal macrophages, immunostaining of lesional macrophages for the expression of iNOS demonstrated fewer M1 macrophages in lesions of IL-13-injected mice (Fig 4B). Moreover, immunostaining for the expression of two different M2 markers, Ym-1 and mannose receptor (MR, CD206), demonstrated significantly higher numbers of M2 macrophages in lesions of IL-13-injected mice, despite an overall decreased macrophage content (Fig 4C and Fig S5B of Supporting Information, respectively). These changes in macrophage activation states resulted in a significantly increased M2:M1 ratio in lesions of IL-13-treated mice (Fig 4D).

In summary, we could demonstrate that the decreased lesional macrophage content was paralleled by the induction of M2 macrophages and concomitantly the reduction of M1 macrophages in atherosclerotic lesions by exogenous administration of IL-13.

### Alternatively activated macrophages by IL-13 show higher clearance of OxLDL *in vitro*

**Figure 5:**
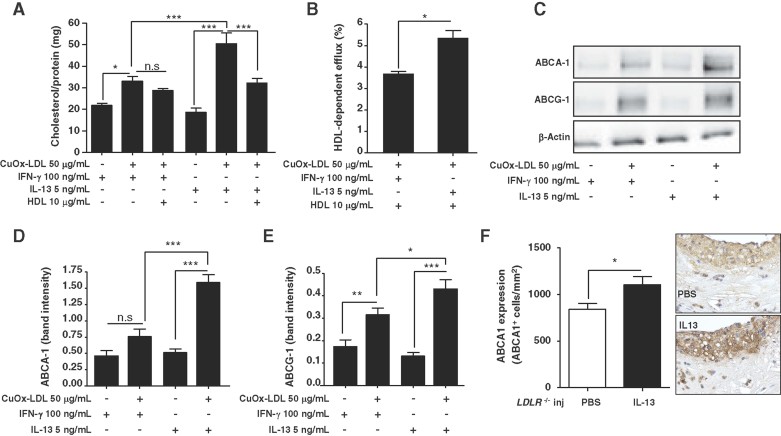
Alternatively activated macrophages (M2) exhibit increased clearance of OxLDL *in vitro* **A.** Thioglycollate-elicited macrophages were stimulated with IFN-γ or IL-13 into classically (M1) or alternatively (M2) activated macrophages, respectively, and then incubated with CuOx-LDL for 24 h to generate foam cells. Increased cellular cholesterol levels in M2-derived foam cells are reduced in the presence of HDL. M1 and M2 macrophages were incubated with CuOx-LDL in the absence or presence of HDL 10 µg/ml. Lipids were extracted from cell lysates and total cholesterol and protein were measured. Data are shown as mean ± SEM values of two independent experiments performed in quadruplicates and represent mg cholesterol/mg protein. *p = 0.04, ***p = 0.0001.**B.** Increased HDL-dependent cholesterol efflux by M2-derived foam cells. M1 and M2 macrophages were incubated with CuOx-LDL plus 1 µM of [^3^H]-cholesterol and HDL-dependent efflux was assayed as described in Materials and Methods Section. Data represent percentages of HDL-dependent efflux/total efflux. *p = 0.010, *t*-test.**C.** Increased ABCA1 and ABCG1 expression in M2-derived foam cells. Shown is a representative Western blot for the presence of ABCA1, ABCG1 and β-actin in lysates of cells that were treated as indicated.**D,E.** The quantification of the band intensity of ABCA1 (**D**) and ABCG1 (**E**) related to β-actin. *p = 0.04, **p = 0.0075, ***p = 0.0001. All data in (**B**–**E**) are shown as mean ± SEM values of three independent experiments performed in triplicates.**F.** Increased ABCA1 expression in lesions of *LDLR*^−/−^ mice injected with IL-13. *LDLR*^−/−^ mice were fed an atherogenic diet for 16 weeks and received biweekly intraperitoneal injections with PBS (*n* = 11) or IL-13 (*n* = 13) during the last 5 weeks. Sections were stained with an antibody against ABCA1 and values represent the numbers of ABCA1^+^ cells/mm^2^ of total lesion area, *p = 0.031. Data are shown as mean ± SEM values of all mice of each group. Images show representative ABCA1 staining. Original magnification 400×.

To study potential effects of differentially activated macrophages in atherogenesis, we evaluated the ability of IFN-γ and IL-13 to modulate macrophage foam-cell formation by promoting either classical (M1) or alternative (M2) macrophage activation, respectively. Thioglycollate-elicited macrophages were stimulated with either IFN-γ or IL-13, and successful differentiation into M1 or M2 macrophages was confirmed by the expression of iNOS (M1) and Arg-1 (M2), respectively (Fig S6A of Supporting Information). Differentially activated macrophages were then incubated with copper-oxidized LDL (CuOx-LDL) to induce foam-cell formation. Subsequent analyses of these cells revealed an increase of total cellular cholesterol content in foam-cell cultures derived from IFN-γ-stimulated macrophages and to a significantly greater extent in macrophages stimulated with IL-13 (Fig 5A). An increased uptake of CuOx-LDL was further confirmed by Oil Red O (ORO) staining, which revealed a higher percentage of ORO^+^ cells in IL-13-activated macrophages compared to IFN-γ-activated macrophages (Fig S6B of Supporting Information). Consistent with that, IL-13 stimulation resulted in increased expression of the scavenger receptor CD36, which was further induced after CuOx-LDL loading (Fig S6C of Supporting Information). The expression of scavenger receptor A-1 (SRA-1) and LOX-1 was not different between the two differentially activated macrophage foam cells (Fig S6D and S6E of Supporting Information). Addition of HDL to the foam-cell cultures significantly reduced the increased cellular cholesterol only in IL-13-activated macrophage cultures, thereby abrogating the increased foam-cell formation (Fig 5A). These data suggested a higher cholesterol efflux capacity of IL-13-activated compared to IFN-γ-activated foam cells.

To further test this hypothesis, we performed *in vitro* cholesterol efflux assays using equal amounts of [^3^H]-cholesterol, which demonstrated a significantly higher HDL-dependent efflux of foam-cell cultures derived from IL-13-stimulated macrophages compared to IFN-γ-stimulated macrophages (Fig 5B). Moreover, to evaluate the ability of IL-13 to directly increase cholesterol efflux in macrophage foam cells, macrophages were first loaded with CuOx-LDL plus [^3^H]-cholesterol and then stimulated with IFN-γ and IL-13, respectively. Importantly, HDL-dependent efflux was significantly increased in IL-13-stimulated foam cells using this experimental setup as well (Fig S7 of Supporting Information). We therefore investigated the expression levels of the two most important transporters responsible for cholesterol efflux in macrophages, ATP-binding cassette A1 (ABCA1) and G1 (ABCG1), by immunoblotting and found that upon stimulation with CuOx-LDL the expression of both ABC transporters was significantly up-regulated in foam cells derived from IL-13-activated macrophages compared to IFN-γ-activated foam cells (Fig 5C–E) and non-activated foam cells (unpublished observation). This was also confirmed by quantitative PCR on the messenger RNA (mRNA) level (Fig S8A and S8B of Supporting Information). In contrast, IFN-γ-activated macrophages exhibited only a significant up-regulation of ABCG1 protein following CuOx-LDL stimulation, albeit to a lesser degree than IL-13-activated macrophages (Fig 5E). Consistent with that, IL-13 stimulation also resulted in the increased expression of the nuclear receptor LXRα, which is the main transcription factor controlling ABCA1 and G1 expression (Fig S8C of Supporting Information).

To correlate these *in vitro* findings with effects of IL-13 on macrophage function *in vivo*, lesions of cholesterol-fed *LDLR*^−/−^ mice that received either IL-13 or PBS (see Fig 1) were analyzed for ABCA1 expression by immunohistochemistry. Remarkably, atherosclerotic lesions of IL-13-treated mice showed a significantly higher number of ABCA1 expressing cells compared to lesions from control mice (Fig 5F).

Taken together, our data demonstrate that macrophages alternatively activated by IL-13 have an overall increased capacity of OxLDL clearance, as they display increased uptake as well as increased cholesterol efflux capacities without enhancing foam-cell formation. This should result in a more efficient removal of pro-inflammatory OxLDL and consequently a less inflammatory environment in atherosclerotic lesions.

### IL-13 deficiency accelerates atherosclerosis

**Figure 6:**
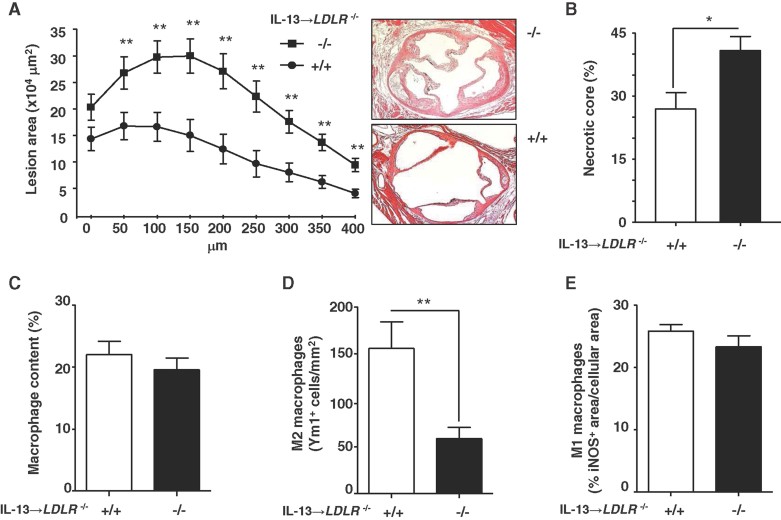
Increased atherosclerosis in *IL-13*-deficient *LDLR*^−/−^ mice *LDLR*^−/−^ mice were reconstituted with bone marrow from either *IL-13*^+/+^ mice (*n* = 12) or *IL-13*^−/−^ mice (*n* = 14) and fed an atherogenic diet for 16 weeks. Increased extent of atherosclerotic lesion size in cross-sections of the aortic origin in mice reconstituted with *IL-13*^−/−^ bone marrow. Values represent µm^2^/section throughout the entire aortic origin, (400 µm). **p = 0.0023. Images show representative H&E stains. Original magnification 50×.Increased necrotic core area in lesions of recipients of *IL-13*^−/−^ bone marrow. Values represent percentages of necrotic core area/total lesion area. *p = 0.016.Lesional macrophage content between recipients of *IL-13*^−/−^ or *IL-13*^+/+^ bone marrow. Sections were stained with the macrophage-specific anti-mac-3 antibody and values represent the percentages of mac-3^+^ area/cellular lesion area.Decreased lesional M2 macrophage content in lesions of recipients of *IL-13*^−/−^ bone marrow. Sections were stained with an antibody against Ym-1, which is specifically expressed by M2 macrophages and values represent number of Ym-1^+^ cells/mm^2^ of cellular lesion area. **p = 0.0043, Mann–Whitney test.Relative lesional M1 macrophage content between recipients of *IL-13*^−/−^ or *IL-13*^+/+^ bone marrow. Sections were stained with an antibody against iNOS, which is specifically expressed by M1 macrophages and values represent the percentages of iNOS^+^ area/cellular lesion area. All data are shown as mean ± SEM values of all mice of each group.

Finally, to demonstrate the role of IL-13 in the development of atherosclerotic lesions, we transplanted lethally irradiated *LDLR*^−/−^ mice with bone marrow from either *IL-13*^+/+^ or *IL-13*^−/−^ mice. Four weeks after bone marrow transplantation (BMT) and successful replenishment, mice were switched to an atherogenic diet for the subsequent 16 weeks to induce atherosclerosis (Fig S9 of Supporting Information). At time of sacrifice, the two groups of mice were not different regarding body weight, TC and TG levels (Table SI of Supporting Information). Importantly, cross-sectional analyses of lesions in the aortic origin revealed significantly accelerated atherosclerosis with almost two times larger lesions in *IL-13*^−/−^ bone marrow recipients, indicating a protective role of IL-13 in atherogenesis (Fig 6A and Table SI of Supporting Information). In addition, *en face* analyses also showed a trend towards increased lesion formation in recipients of *IL-13*^−/−^ bone marrow (Table SI of Supporting Information).

Lesions of *IL-13*^−/−^ bone marrow chimeras displayed increased necrotic core formation, consistent with advanced plaque progression (Fig 6B). Nevertheless, the relative macrophage content was equivalent between the two groups (Fig 6C) with significantly less M2 macrophages in lesions of *IL-13*^−/−^ bone marrow chimeras (Fig 6D). The predominant M1 macrophage areas were similar between the two groups (Fig 6E).

**Figure 7:**
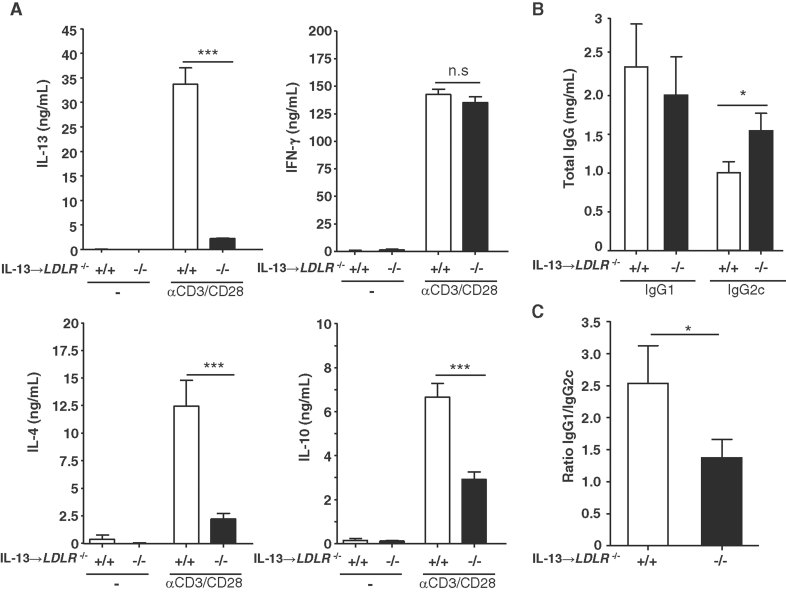
Effect of *IL-13* deficiency on splenic cytokine production and antibody isotype levels *LDLR*^−/−^ mice were reconstituted with bone marrow from either *IL-13*^+/+^ mice (*n* = 12) or *IL-13*^−/−^ mice (*n* = 14) and fed an atherogenic diet for 16 weeks. At time of sacrifice, spleens and blood were collected from all mice. Recipients of *IL-13*-deficient bone marrow show a decreased production of Th2 cytokines (IL-13, IL-4 and IL-10) but not IFN-γ by splenocytes stimulated with anti-CD3/CD28 *in vitro*. Data are presented as ng/ml cytokine of splenocyte cultures ***p = 0.0001.Increased levels of total IgG2c antibodies in sera of *IL-13*-deficient *LDLR*^−/−^ mice. Data are presented as mg/ml of indicated serum IgG isotypes. *p = 0.029.Decreased ratio of IgG1:IgG2c antibodies in *IL-13*-deficient *LDLR*^−/−^ mice. *p = 0.04. All data are shown as mean ± SEM values of all mice of each group.

To investigate potential immunological differences paralleling this increased lesion formation, total splenocytes of mice from both groups were stimulated with anti-CD3 and anti-CD28 for 72 h to induce maximal T-cell activation *in vitro*. As expected, splenocytes from *IL-13*^−/−^ *LDLR*^−/−^ bone marrow chimeras produced only minimal amounts of IL-13. Moreover, the production of IL-4 and IL-10, but not IL-5 and IFN-γ were significantly diminished in these mice (Fig 7A and Table SI of Supporting Information). This selective decrease in Th2 cytokine production was also reflected by a significant increase in Th1-dependent IgG2c antibodies in serum of the *IL-13*^−/−^ *LDLR*^−/−^ bone marrow chimeric mice, while total IgG1 antibody levels were not different (Fig 7B and Table SII of Supporting Information). IgM levels were similar between both groups (Table SI of Supporting Information). These changes in T-cell-dependent IgG levels resulted in a significantly decreased IgG1:IgG2c ratio indicating an overall Th1-biased response (Fig 7C). Similar results were obtained for MDA-LDL-specific IgG1 and IgG2c titers, respectively (Fig S10A of Supporting Information). Importantly, the numbers of splenic T cells and B cells (including B1 and B2 cells) were not different between the two groups (Table SI of Supporting Information). Consistent with an inherent Th1 bias, non-atherosclerotic *IL-13*-deficient mice that were used as bone marrow donors were also found to display decreased IgG1 and increased IgG2c levels compared to wild type controls (Fig S10B of Supporting Information).

These data indicate that IL-13 deficiency results in an overall pro-inflammatory environment that inhibits alternative activation of lesional macrophages and promotes atherosclerotic lesion development.

## DISCUSSION

In the current study, we demonstrate a previously unrecognized atheroprotective role of IL-13 in murine models of atherogenesis. It was assumed that IL-13 would have a similar pro-atherogenic role in atherosclerosis as IL-4, because both cytokines share similar functions by engaging the same receptor complexes. Our data now point to a differential role in atherosclerosis, which can be explained by unique functions of IL-4 and IL-13 as a consequence of the exclusive engagement of the alternative receptors IL-4Rα/γc and IL-13Rα2, respectively, or by differences in ligand affinity for the same IL-4Rα/IL-13Rα1 receptor complex (Kelly-Welch et al, 2003; LaPorte et al, 2008). This is exemplified by the ability of only IL-4 to differentiate naïve CD4^+^ T cells into Th2 cells (Seder & Paul, 1994) or the non-redundant role of IL-13 in parasite expulsion, allergic inflammation and asthma (Bancroft et al, 1998; Grunig et al, 1998; Liang et al, 2011).

Specifically, we found that cholesterol-fed chimeric *IL-13*^−/−^ *LDLR*^−/−^ mice develop nearly twofold larger lesions in the aortic origin than *LDLR*^−/−^ mice that were reconstituted with wild type bone marrow. This pro-atherogenic effect of IL-13 deficiency was accompanied by an overall Th1-biased immune phenotype, as judged by cytokine release of stimulated splenocyte cultures and Th1/Th2-dependent IgG isotype levels in sera of these mice. Our data are consistent with the known impairment in Th2-cell development in *IL-13*-deficient mice (McKenzie et al, 1998a). Likely this overall shift in the immune response contributed in part to the pro-atherogenic effect observed. The profound effects of IL-13 deficiency on atherosclerotic lesion formation discouraged detailed analyses of plaque morphology, as a meaningful interpretation of potential differences in content of lesions of different size and stage is virtually impossible.

To study the atheroprotective effect of IL-13 directly, we examined the impact of exogenous IL-13 administration on established atherosclerotic lesions in cholesterol-fed *LDLR*^−/−^ mice. To avoid hepatic fibrosis (Wynn, 2008), we chose a low dose of IL-13 administration to achieve only three times higher serum levels than found in atherosclerotic mice. Importantly, this interventional strategy did not result in an alteration of the Th1/Th2 phenotype of the immune response. Nevertheless, we discovered that IL-13 administration resulted in significantly increased lesional collagen content, which is consistent with the known strong pro-fibrotic role of IL-13 (Wynn, 2008), as well as significantly decreased lesional macrophage content. These alterations in plaque morphology are strongly reminiscent of changes that were reported to occur as a result of lesion regression induced by lowering serum cholesterol or increasing serum HDL in mice (Reis et al, 2001; Rong et al, 2001; Williams et al, 2008). Of interest, the changes in our model of IL-13 administration occurred without changes in serum cholesterol levels. It remains to be shown whether IL-13 is also mechanistically involved in atherosclerotic lesion stabilization during lipid lowering or active lesion regression.

In this regard it is important to point out that Fisher and colleagues recently reported that regression of atherosclerotic lesions is associated with the up-regulation of markers of alternative macrophage activation (M2) (Feig et al, 2011a, b; Rayner et al, 2011). M2 macrophages have been documented in murine and human lesions (Bouhlel et al, 2007; Chinetti-Gbaguidi et al, 2011; El Hadri et al, 2012; Feig et al, 2012; Khallou-Laschet et al, 2010). We now show that IL-13 administration also induces relative and absolute increases in M2 macrophages in cholesterol-fed *LDLR*^−/−^ mice and that the same lesions that show increased collagen and a decreased macrophage content have significantly increased numbers of alternatively activated macrophages and furthermore, concomitantly decreased numbers of classically activated macrophages (M1). Because our quantitative assessment of lesional macrophages suggests the presence of still ‘uncommitted’ macrophages (*i.e.* iNOS^−^ and Ym-1^−^), we believe that IL-13 primarily acts on this particular population of existing macrophages. In contrast, lesions of cholesterol-fed chimeric *IL-13*^−/−^ *LDLR*^−/−^ mice displayed decreased numbers of M2 macrophages. Although this dual classification pattern of macrophages has been considered overly simplistic, it helped characterizing macrophage heterogeneity and plasticity within atherosclerotic plaques (Feig et al, 2011a, b; Khallou-Laschet et al, 2010; Stoger et al, 2010). Our data show that the macrophage phenotype in atherosclerotic lesions can be modulated by IL-13 independent of cholesterol-lowering.

We also addressed the functional consequences of increased numbers of M2 macrophages with respect to uptake of OxLDL and cholesterol efflux, rate limiting steps in foam-cell formation during atherogenesis (Steinberg & Witztum, 2010). Using murine primary macrophages differentially activated with either IL-13 or IFN-γ, respectively, to model potential extremes of cytokine exposure inside the plaques, we observed an increased capacity of IL-13-stimulated macrophages to take up OxLDL. This is consistent with previous studies showing that M2 macrophages exhibit increased expression of scavenger receptor CD36 and possess higher phagocytic activity (Berry et al, 2007; Gordon & Martinez, 2010). Previously, IL-4/IL-13 stimulation has been shown to activate PPARγ, leading to upregulation of CD36 through the generation of endogenous ligands in murine and human macrophages (Huang et al, 1999; Rey et al, 1998), which might be predicted to lead to enhanced foam-cell formation. Indeed, we could also demonstrate increased expression of CD36, but not SRA-1 or LOX-1, in IL-13-activated macrophage foam cells. We also found that OxLDL-loaded IL-13-activated macrophages exhibited a higher cholesterol-efflux capacity and had increased expression of ABCA1 and ABCG1 compared to IFN-γ-activated macrophages, resulting in no net increase in cholesterol accumulation. Our data is supported by a previous report demonstrating decreased ABCA1 expression and cholesterol-efflux of IFN-γ-treated murine foam cells compared to unstimulated foam cells (Panousis & Zuckerman, 2000). If translatable to the *in vivo* situation, our data suggest an enhanced ability of IL-13-stimulated M2 type macrophages to clear OxLDL from the extracellular environment and efficiently promote efflux of free cholesterol via ABCA1/G1 pathways without enhancing foam-cell formation—a protective response that would be desirable in a lesional macrophage. In fact, we did find increased expression of ABCA1 in lesions of IL-13-treated mice. It is noteworthy that Chinetti-Gbaguidi et al recently reported that IL-4-activated human monocyte-derived macrophages are less prone to foam-cell formation, although they found lower efflux capacity and increased cholesterol esterification in these cells compared to untreated cells. This comparison to ‘neutral’ monocytes and differences between IL-4 and IL-13 may explain the discrepancies (Chinetti-Gbaguidi et al, 2011).

A prominent consequence of IL-13 administration was decreased recruitment of monocytes to carotid arteries of atherosclerotic *ApoE*^−/−^ mice, whereas no effect on lesional macrophage egression was observed. The diminished recruitment seems to be largely a consequence of decreased endothelial VCAM-1 expression, which we observed in atherosclerotic mice treated with IL-13. A mechanistic role for VCAM-1 is further supported by the fact that only recruitment of monocytes, but not neutrophils, was affected by the IL-13 intervention. Furthermore, no difference between the recruitment of nonclassical Ly6C^lo^ or classical Ly6C^hi^ monocytes was observed, which is typically dependent on the expression of specific chemokines (Combadiere et al, 2008; Ingersoll et al, 2011; Tacke et al, 2007). In agreement with that, we did not observe an alteration of CCL2 presentation by endothelial cells in the carotid arteries of atherosclerotic *ApoE*^−/−^ mice injected with IL-13. Finally, in analogy to the parallels with lesion regression discussed above, Potteaux et al recently reported that decreased monocyte recruitment during lesion regression was also associated with decreased endothelial VCAM-1 expression (Potteaux et al, 2011). Endothelial VCAM-1 expression is a key event during atherosclerotic lesion formation (Cybulsky et al, 2001; Dansky et al, 2001). However, it is unlikely that IL-13 administration had a direct effect on VCAM-1 expression, as a previous study demonstrated that IL-13 in fact promoted the up-regulation of VCAM-1 on activated-endothelial cells *in vitro* (Bochner et al, 1995; Woltmann et al, 2000). It is known that VCAM-1 is strongly induced by OxLDL in endothelial cells *in vitro* and at lesion prone-sites even before the appearance of visible lesions (Cybulsky & Gimbrone, 1991; Khan et al, 1995). Therefore, considering our data, we would speculate that a more likely explanation for the decreased VCAM-1 expression is a reduced intimal content of OxLDL as a consequence of enhanced IL-13-induced M2 macrophage-mediated clearance.

In conclusion, our data indicate a key role for IL-13 in halting the progression of atherogenesis and promoting plaque stabilization. We provide evidence that IL-13 leads to decreased VCAM-1 mediated monocyte recruitment to atherosclerotic lesions, enhanced phenotypic modulation towards the reparative and atheroprotective M2 phenotype, and enhanced collagen deposition. Even in the absence of decreased plasma cholesterol levels, the changes induced by IL-13 are strongly reminiscent of effects seen during plaque regression in response to cholesterol lowering. Thus, our findings identify a potential new target for the prevention and treatment of atherosclerosis.

## MATERIALS AND METHODS

An expanded Materials and Methods Section is available in the Supporting Information.

### Animal and intervention studies

LDL receptor-deficient mice (*LDLR*^−/−^) and C57BL/6J were from The Jackson Laboratories (Bar Harbor, Maine, USA); *IL-13*^−/−^ mice were a kind gift of Dr. Thomas Wynn (NIAID/NIH, Bethesda, USA). All mice were on a C57BL/6J background (tenth generation) and were bred in-house. All experimental protocols were approved by the institutional animal experimentation committee and the Austrian Ministry of Science.

**The paper explained**

PROBLEM:

Atherosclerosis is a chronic inflammatory disease of the vessel wall and the underlying cause of heart attacks and a majority of strokes. Atherosclerotic lesions are characterized by infiltrating macrophages that take up accumulating lipids resulting in the formation of pro-inflammatory foam cells. Innate and adaptive immunity modulate the development and progression of atherosclerotic lesions, which provides potential therapeutic targets in addition to existing cholesterol lowering strategies. The functions of a number of cytokines, including the pro-inflammatory effects of IFN-γ, are well established. However, the role of interleukin-13 (IL-13) is still unknown.

RESULTS:

In our study, we show that IL-13 administration limits the recruitment of macrophages to and promotes collagen production in established lesions of atherosclerosis-prone mice. These effects are paralleled by IL-13-dependent changes in the activation states of plaque macrophages, and we could show that IL-13-activated macrophages (M2) possess more efficient, beneficial lipid handling capacities compared to IFN-γ-activated macrophages (M1) *in vitro*. Importantly, atherosclerosis-prone mice that were incapable of secreting IL-13 by hematopoietic cells developed significantly bigger and more advanced atherosclerotic plaques.

IMPACT:

Our findings indicate a protective role for IL-13 in atherosclerosis. Remarkably, IL-13 has the capacity to alter plaque morphology in the presence of high serum cholesterol levels towards more stable, less vulnerable plaques. Because IL-13 leads to alternative macrophage activation in atherosclerotic lesions, and these macrophages have anti-atherogenic properties, our data identify macrophage polarization by IL-13 as novel point for therapeutic intervention.

Bone marrow transplantation studies were performed as previously described (Binder et al, 2004). Thirty 8-week-old male *LDLR*^−/−^ mice were given a single dose of 9-Gy lethal irradiation and irradiated mice were injected intravenously with 2 × 10^6^ bone marrow cells harvested from either *IL-13*^−/−^ (*n* = 15) or *IL-13*^+/+^ (*n* = 15) mice. Mice were fed regular chow diet for 4 weeks after BMT to allow for bone marrow reconstitution and then switched to an atherogenic diet containing 21% fat and 0.2% cholesterol (TD88137, Ssniff Spezialdiäten GmbH, Soest, Germany) for an additional 16 weeks to induce lesion formation.

For the atherosclerosis intervention study, twenty-four 12-week-old female *LDLR*^−/−^ mice were fed an atherogenic diet (Ssniff) for a total of 16 weeks to induce lesion formation. At week 11, mice were divided randomly into two groups and injected intraperitoneally with PBS (*n* = 11) or IL-13 (50 ng/mouse R&D systems, Minneapolis, Minnesota, USA; *n* = 13) twice per week for the following remaining 5 weeks.

### Evaluation and phenotypic analysis of atherosclerotic lesions

The extent of atherosclerosis was determined in a blinded fashion in *en face* preparations of the entire aorta, as well as in cross sections through the aortic origin, by computer-assisted image analysis as previously described (Binder et al, 2004). Lesion phenotype was determined by the content of collagen, size of necrotic core area, and the presence of macrophages, smooth-muscle cells, T cells, classically activated (M1) macrophages, alternatively activated (M2) macrophages, and ABCA1 expressing cells in lesions of equal size. For the collagen content, sections were stained with Sirius Red, and for the assessment of necrotic cores, sections were stained with a modified elastic-trichrome stain. For the presence of macrophages, smooth-muscle cells, T cells, M1 macrophages, M2 macrophages and ABCA1 expressing cells immunohistochemistry was performed using antibodies against mouse Mac-3 (BD-Biosciences Pharmingen, San Diego, California, USA), smooth-muscle cell actin (Sigma-Aldrich), CD3 (DAKO, Glostrup, Denmark), iNOS (ABCAM, Cambridge, UK), CD206 (BioLegend, San Diego, California, USA), Ym1/2 (a kind gift of Dr. Shioko Kimura, NIH/NCI, Bethesda, USA) and ABCA1 (Novus Biological, Littleton, Colorado, USA). The photographed images were analyzed using ImageJ 1.41 software.

### Macrophage foam-cell assays

For all *in vitro* experiments, C57BL/6J mice, 12–16 weeks of age, were injected intraperitoneally with 2 ml of 3% thioglycollate (Difco, Thermo Fischer Scientific). After 3 days, thioglycollate-elicited macrophages were harvested and differentiated into classically activated macrophages (M1) with 100 ng/ml IFN-γ (R&D systems) or alternatively activated macrophages (M2) with 5 ng/ml IL-13 (R&D systems) for 16 h. Following differentiation, cells were stimulated with 50 µg/ml CuOx-LDL in the presence or absence of 10 µg/ml of HDL in full culture medium containing 1% mouse serum for 24 h to induce foam-cell formation. Subsequently, cholesterol efflux, RNA/protein isolation and cellular cholesterol quantification experiments were performed as described in the Supporting Information. For primer sequences see Table SII of Supporting Information.

### Monocyte labeling and macrophage egression assessment

Twenty-two male, 8-week-old, *ApoE*^−/−^ mice (C57BL/6J background) were fed a standard western diet containing 21% fat and 0.2% cholesterol (Altromin Spezialfutter GmbH & Co.KG, Lage, Germany) for 6 weeks to induce lesion formation. At week 3, circulating classical Ly6C^hi^ monocytes were labelled by intravenous (i.v) injection of 1 µm Fluoresbrite green fluorescent (YG) plain microspheres (Polysciences Inc., Warrington, Pennsylvania, USA), 24 h after clodronate-liposome depletion of monocytes. Latex-beads^+^monocytes were allowed to accumulate within atherosclerotic plaques for 1 week. At week 4, mice were divided into three groups. One group was sacrificed for the quantification of bead and macrophage (mac-2^+^ cells) content (baseline, *n* = 7); the remaining two groups received biweekly intraperitoneal injections of either PBS (*n* = 7) or IL-13 (50 ng/mouse, R&D systems *n* = 8) for the remaining 2 weeks. All animal experiments were approved by the local ethical committee (Regierung von Oberbayern). Macrophage emigration from atherosclerotic lesions was analyzed as described previously (Potteaux et al, 2011).

### Intravital microscopy

Sixteen male, 8-week-old, *Cx3cr1*^gfp/wt^ *ApoE*^−/−^ mice (C57BL/6J background, for leukocyte endothelial interactions) and 16 male, 8-week-old, *ApoE*^−/−^ mice (C57BL/6J background, for endothelial adhesion molecules expression) were fed an atherogenic diet containing 21% fat and 0.2% cholesterol (Altromin) for 6 weeks to induce lesion formation. At week 4, they were randomly divided into two groups and injected intraperitoneally with PBS (*n* = 8) or IL-13 (50 ng/mouse, R&D systems, *n* = 8) twice per week for the remaining 2 weeks. At the end of this period, mice were anaesthetized with ketamine/xylazine and leukocyte endothelial interactions (Drechsler et al, 2010) and expression of endothelial adhesion molecules (Engel et al, 2011) were analyzed by intravital microscopy of the left carotid artery as described previously. To permit discrimination of phagocyte subsets a PE-conjugated antibody to Gr1 (eBioscience) was introduced via an intravenous catheder 5 min prior to recording. All animal experiments were approved by the local ethical committee (Regierung von Oberbayern).

### Statistical analysis

Statistical analyses were performed by unpaired Student *t*-test for all results of *in vivo* studies and by one-way analysis of variance (with Bonferoni post-test analysis) for all *in vitro* data (unless indicated differentially) to determine statistical significance between the groups. Data are presented as mean ± SEM and *p* < 0.05 was considered significant.

## Supplementary material

Detailed facts of importance to specialist readers are published as ”Supporting Information”. Such documents are peer-reviewed, but not copy-edited or typeset. They are made available as submitted by the authors.
