# Supplementary material for: Interleukin-13 protects from atherosclerosis and modulates plaque composition by skewing the macrophage phenotype
Source: EMBO Mol Med. 2012 Oct 2;4(10):1072–86. doi: 10.1002/emmm.201201374 (PMC3491837; doi:10.1002/emmm.201201374)
Supplement: Supplementary file 1 [file emmm0004-1072-SD1.pdf]

EMBO
Molecular Medicine

Manuscript EMM-2012-01374

Interleukin-13 protects from atherosclerosis and modulates plaque composition by skewing the macrophage phenotype

Larissa Cardilo-Reis, Sabrina Gruber, Sabine M. Schreier, Maik Drechsler, Nikolina Papac-Milicevic, Christian Weber, Oswald Wagner, Herbert Stangl, Oliver Soehnlein, and Christoph J. Binder

Corresponding author: Christoph Binder, Medical Univeristy of Vienna / Center for Molecular Medicine

Review timeline:

Submission date:	14 March 2012
Editorial Decision:	10 April 2012
Revision received:	16 July 2012
Accepted:	03 August 2012

Transaction Report:

(Note: With the exception of the correction of typographical or spelling errors that could be a source of ambiguity, letters and reports are not edited. The original formatting of letters and referee reports may not be reflected in this compilation.)

1st Editorial Decision 10 April 2012

Thank you for the submission of your manuscript "Interleukin-13 protects from atherosclerosis and modulates plaque composition by skewing the macrophage phenotype" to EMBO Molecular Medicine. We have now heard back from the three referees whom we asked to evaluate your manuscript. You will see that they find the topic of your manuscript potentially interesting. However, they also raise significant concerns on the study, which should be addressed in a major revision of the manuscript.

In particular, reviewer #1 highlights that cholesterol uptake routes should be assessed in addition to the efflux pathways. Importantly, reviewer #3 also notes concerns regarding assessment of cholesterol efflux from treated macrophages. In addition, reviewer #1 points out that absolute numbers for macrophages should be provided while reviewer #3 notes that macrophage egress should be differentiated from decreased macrophage recruitment.

On a more editorial note, please see our Instructions to Authors on statistical analysis and mention the actual P value for each test (not merely 'significant' or 'P < 0.05') (http://onlinelibrary.wiley.com/journal/10.1002/(ISSN)1757-4684/homepage/ForAuthors.html#data2).

Given the balance of these evaluations, we feel that we can consider a revision of your manuscript if you can convincingly address the issues that have been raised within the space and time constraints outlined below.

Revised manuscripts should be submitted within three months of a request for revision. They will otherwise be treated as new submissions, unless arranged differently with the editor.

I look forward to seeing a revised form of your manuscript as soon as possible.

Yours sincerely,

Editor
EMBO Molecular Medicine

***** Reviewer's comments *****

Referee #1 (Comments on Novelty/Model System):

All experiments seem technically sound. It is the first paper to show a role for IL-13 in regulating M2 macrophages in murine atherosclerosis with phenotypic effects on the plaques. Although previous data on IL-4 induced M2 macrophages in mouse models were not able to detect a clear role for these M2 macrophages, this paper does so for the other M2 inducing cytokine IL-13. It highly contributes to our understanding of inflammatory regulation in atherogenesis. These data also show that it will be very relevant to study Il-13 in human atherosclerosis as well.

Referee #1 (Other Remarks):

The paper by Cardilo-Reis and co-workers investigates the role of IL-13 in murine atherosclerosis. Using a treatment approach and a bone marrow transfer of IL-13 deficient bone marrow they show that IL-13 induces M2 macrophages in the context of atherosclerosis and has anti-atherosclerotic, plaque stabilizing effects. The paper is well written and experimentally sound. It contains important novel data about the role of M1 and M2 macrophages in atherosclerosis and is the first to demonstrate that modulation of alternatively activated macrophages affects atherogenesis.

Major points:

Part of the atherosclerotic phenotype is explained by effects on cholesterol efflux pathways. However, to my opinion the data in figure mainly point towards an effect on uptake pathways. The key effect that is seen in figure 4A is a difference in cholesterol accumulation in response to oxLDL incubation. This is of particular importance because data from another group (Chinetti-Gbaguidi et al. 2011) actually show that IL-4 induced macrophages, accumulate less lipids, show differences in uptake receptors and have reduced ABC expression. Therefore, it will be important to assess the uptake routes as well, by showing uptake assays and gene expression for scavenger receptors. In addition, LXR expression data will be informative. This will complete the picture of IL-13 induced macrophage cholesterol metabolism.

The immuno histochemistry is not completely convincing. Better representative photographs should be shown. The photographs in 4C are an M2 macrophage staining (it is unclear whether this is CD206 or Ym1) but seems to show two macrophage types. One with a clear nuclear staining for the marker (indicated by the arrow) and another population residing in the cap with a clear cytoplasmic staining. Maybe, these pictures are not representative but then other photographs should be shown. Overall, the paper (particularly figure 3) would highly benefit from quantifying absolute cell numbers positive for Mac3, M1 and M2 markers, especially since part of the phenotype is linked to cholesterol accumulation which may affect cell size in the plaque. With absolute cell numbers, changes in M1 and M2 ratio can be assessed more easily.

In the treatment study major effects on collagen were observed. How was collagen affected in the knockout study?

In figure 1A and figure 5A atherosclerotic lesion size is shown in two different ways. Please us one approach for the aortic root.

Minor points:

In the introduction the paper by King et al. is referred to as a recent paper but it is already 5 years old.
The hallmark paper by Bouhlel in Cell Metabolism should be mentioned earlier-on in the paper, in the introduction.
This reviewer would not call a 5 week period a short treatment, unable to affect plaque size. There is ample literature showing effect of 5 weeks or shorter treatments on atherosclerotic plaque size.
The title of figure S2 is wrong, stating that there is a phenotypic difference induced by IL-13, but the data in S2 show no difference.
The photograph of figure 3b (bottom) should be replaced by a better one. This photograph seems overexposed or with less contrast then the top one.

Referee #2 (Comments on Novelty/Model System):

This is an original study that provide novel mechanistic insights into the role of IL-13 in atherosclerosis. The authors used sound murine models of atherosclerosis, and state-of-the-art technology to explore the role of macrophages and T cells. My only concern is that the authors should investigate the role of B cells in more details.

Referee #2 (Other Remarks):

This is an original study that provide novel insights into the role of IL-13 in atherosclerosis. By using different models of atherosclerosis, the authors demonstrated that IL-13-treatment decreased atherosclerosis in apoE-/- mice, whereas transplantation of IL-13 deficient bone marrow to LDLr-/- mice exaggerated atherosclerosis. They also found that Il-13 favored M1/M2 switch in macrophage phenotype and decreased VCAM-1 dependent monocyte adhesion to the endothelium.

Comments

1. page 5. 1st paragraph. What about B cell sub-populations and IgG/IgM antibody levels following IL-13 administration in apoE-/- mice?
2. page 6, 2nd paragraph. How did the authors technically discriminate between GFPhigh and GFPlow monocytes by using intravital microcospy? We antivipate that low GFP levels were barely detectable with this technique.
3. page 9. Information about splenic B cell populations in the BMT model would be of interest.

Referee #3:

In the current work, Cardilo-Reis et al. evaluate the role of IL-13 in atherosclerosis. IL-13 is an interesting cytokine, given that it polarizes macrophages towards an M2 phenotype, which is considered to be anti-inflammatory and anti-atherogenic. Indeed, lack of IL-13 promotes atherogenesis in LDLR-/- mice, while IL-13 administration in hypercholesterolemic LDLR-/- mice with established lesions modulates the plaque morphology to promote lesion resolution. Therefore, the findings reported herein are highly pertinent to cardiovascular research, and of specific interest to the atherosclerosis research community. Importantly, this study points to a novel potential role for IL-13 in the treatment of atherosclerosis, and the key finding is that is may do so in the face of persistent hypercholesterolemia.

Major comments:

1. In the first results section, the authors show a reduction in macrophage recruitment to atherosclerotic lesions, following IL-13 administration.
a. Is IL-13 really modulating recruitment, or could it be promoting egress of macrophages from the lesions?
i. Given the increase in collagen in arterial macrophages, macrophage egress seems probable. This

could be addressed using macrophage trafficking techniques such as fluorescently-labeled beads.
ii. How to explain a reduction in arterial macrophages in response to IL-13 treatment in hypercholesterolemic mice, but comparable arterial macrophage content in lesions of IL-13/LDLR-/- and LDLR-/- mice? Seems inconsistent with a role for IL-13 in macrophage recruitment to the plaque. Wouldn't higher numbers of arterial macrophages in the IL-13/LDLR-/- mice as compared to LDLR-/- mice be expected?

2. In regards to the observed induction of M2 macrophage and concomitant reduction of M1 macrophages, could the authors comment on what they think is happening: are M1 macrophages switching to M2 macrophages (ie regression), or does IL-13 only act on the newly recruited macrophages?
a. If foam cells (M1) are treated in vitro with IL-13, can they be polarized to an M2 phenotype? Or does IL-13 only have this effect on 'neutral' or 'M0' macrophages?

3. In Fig4b, where cholesterol efflux from IFNg- and IL-13-treated macrophages is compared, cholesterol efflux from the two groups likely isn't comparable because the starting specific activity of cholesterol wouldn't be the same in IFN- and IL-13-treated cells, due to the unequal loading of the macrophages from the two groups (ie there is more OxLDL loading in IL-13-primed macrophages compared to IFN-primed macrophages, and consequently the cholesterol label would be more dilute and would likely label a different pool (the lipid droplets) in the IL-13-treated cells as compared to the IFN-treated cells). A better way to directly compare efflux from IFN and IL-13-treated cells would be to first label the macrophages with 3H-cholesterol-OxLDL, to get equal loading, and then assess the effect of IFN and IL-13 treatment on efflux. The treatment of these foam cells would better mimic the situation in vivo, when IL-13 is administered to hypercholesterolemic mice.

4. In Fig 4c, when performing western blots for ABCA1 and ABCG1 expression following OxLDL loading of IFN- and IL-13-treated macrophages, an 'unskewed' or 'neutral' macrophage control (+ or - OxLDL) should be included.
a. Do you expect an intermediate ABCA1 upregulation in 'M0' macrophages as compared to M1 and M2 macrophages, following OxLDL loading?
b. Can IL-13 rescue the impaired ABCA1 upregulation in IFN-primed M1 macrophages, or does IL-13 further increase ABCA1 expression in macrophages pre-loaded with OxLDL?
c. Could lesional macrophage ABCA1 expression be assessed in plaques from IL-13 KO and IL-13-treated mice?

5. The chosen dose of IL-13 administered was fixed to be 3X that of the IL-13 concentration found in the serum of atherosclerotic mice.
a. How does that compare to IL-13 'basal' levels? After 16 weeks on a hypercholesterolemic diet, are IL-13 serum levels reduced in LDLR-/- mice as compared to prior to commencement of the hypercholesterolemic diet?

Minor comments:

1. Which monocyte populations the Ly6Clo and Ly6Chi cells represent, ie. resident versus inflammatory monocytes, should be specified in the results section to facilitate the reader's interpretation of the results.

2. In Fig4A, given that all the 3 doses of HDL tested give the same results, perhaps it is unnecessary to present all 3 - keeping only the 10ug/mL dose, which was used for the efflux in Fig4B, may simplify the message the graph is trying to convey to the reader.

1st Revision - Authors' Response 16 July 2012

***** EMBO - Molecular Medicine *****

***** Reviewer's comments *****

Referee #1:

All experiments seem technically sound. It is the first paper to show a role for IL-13 in regulating M2 macrophages in murine atherosclerosis with phenotypic effects on the plaques. Although previous data on IL-4 induced M2 macrophages in mouse models were not able to detect a clear role for these M2 macrophages, this paper does so for the other M2 inducing cytokine IL-13. It highly contributes to our understanding of inflammatory regulation in atherogenesis. These data also show that it will be very relevant to study IL-13 in human atherosclerosis as well.

The paper by Cardilo-Reis and co-workers investigates the role of IL-13 in murine atherosclerosis. Using a treatment approach and a bone marrow transfer of IL-13 deficient bone marrow they show that IL-13 induces M2 macrophages in the context of atherosclerosis and has anti-atherosclerotic, plaque stabilizing effects. The paper is well written and experimentally sounds. It contains important novel data about the role of M1 and M2 macrophages in atherosclerosis and is the first to demonstrate that modulation of alternatively activated macrophages affects atherogenesis.

Major points:

1) Part of the atherosclerotic phenotype is explained by effects on cholesterol efflux pathways. However, to my opinion the data in figure 4 mainly point towards an effect on uptake pathways. The key effect that is seen in figure 4A is a difference in cholesterol accumulation in response to oxLDL incubation. This is of particular importance because data from another group (Chinetti-Gbaguidi et al. 2011) actually show that IL-4 induced-macrophages accumulate less lipids, show differences in uptake receptors and have reduced ABC expression. Therefore, it will be important to assess the uptake routes as well, by showing uptake assays and gene expression for scavenger receptors. In addition, LXR expression data will be informative. This will complete the picture of IL-13 induced macrophage cholesterol metabolism.

We thank the Reviewer for careful reading of our manuscript and raising these important points. Indeed, we believe that both alterations of the uptake and the efflux pathways contribute to the functional consequences in lipid accumulation we see in response to IL-13. As suggested by the Reviewer, we have performed CuOx-LDL uptake assays and analyzed scavenger receptors expression in differentially activated macrophages (i.e. IFNγ and IL-13, respectively) in the absence of HDL. Oil-Red O staining of these macrophages showed an increased percentage of foam-cells in IL-13-activated macrophages, which is consistent with an increased uptake. These new data have now been added as **Supplemental Figure**

S6 B of the Support information in the revised version of this manuscript. They are also consistent with the data on cholesterol accumulation reported in Figure 5A (former 4A) of the main manuscript. Moreover, expression of CD36 was found to be significantly increased in IL-13-activated macrophages compared to IFNγ-activated macrophages and further increased upon incubation with CuOx-LDL (see **Supplemental Figure S6 C** of the Support information of the revised manuscript). In contrast, SRA-1 and LOX-1 expression were not different between the two differentially activated macrophages in the absence or presence of CuOx-LDL (see **Supplemental Figure S6 D&E** of the Support information of the revised manuscript). Because we also report that IL-13-activated foam cells have an increased cholesterol efflux capacity (new Figure 5), we further corroborated our data on the increased expression of ABCA1 and ABCG1 by assessing the expression of LXRs in response to IL-13, as suggested by this Reviewer. Consistently, LXRα expression (but not LXRβ) was induced by IL-13 and further increased upon CuOx-LDL loading (see **Supplemental Figure S8 C&D** of the Support information of the revised manuscript). Thus, IL-13 results in an increased uptake of OxLDL as a result of increased CD36 expression, and at the same time in an increased cholesterol efflux capacity. Together this results in more efficient clearance of OxLDL by IL-13-activated macrophages.

As pointed out by this Reviewer our findings differ from those reported by Chinetti-Gbaguidi *et al*. 2011, who compared the lipid handling between human peripheral blood monocytes that were either stimulated with IL-4 (M2) or left untreated (resting monocytes; RM). We believe that in addition to the potential differences between IL-4 and IL-13, and the cell types used, these differences could be explained by the fact that the control cells (RM) used in the paper by Chinetti-Gbaguidi represent cells with an undefined (uncontrolled) state of activation. In contrast, our data directly compare two different types of polarized macrophages, which we believe reflect activation states that are induced in atherosclerotic lesions *in vivo*.

2) The immunohistochemistry is not completely convincing. Better representative photographs should be shown. The photographs in 4C are an M2 macrophage staining (it is unclear whether this is CD206 or Ym1) but seems to show two macrophage types. One with a clear nuclear staining for the marker (indicated by the arrow) and another population residing in the cap with a clear cytoplasmic staining. Maybe, these pictures are not representative but then other photographs should be shown. Overall, the paper (particularly figure 3) would highly benefit from quantifying absolute cell numbers positive for

Mac3, M1 and M2 markers, especially since part of the phenotype is linked to cholesterol accumulation which may affect cell size in the plaque. With absolute cell numbers, changes in M1 and M2 ratio can be assessed more easily.

We apologize for not being clear in the presentation of our immunohistochemistry data, and we agree that it is difficult to judge these given the magnifications we have supplied in the original submission. As suggested by the Reviewer, we have now quantified total macrophage (mac-3), M1 (iNOS) and M2 (Ym-1) macrophage numbers. We are providing new better photographs of these stains (**Figure 1C** and **Figure 4 B&C** of the revised manuscript) and are enclosing a higher magnification of these for the attention of this Referee (see below – Figure R1). These pictures clearly show cytoplasmatic staining for all markers. Quantification of mac-3^+ cells confirmed a significantly decreased number of macrophages/mm^2 ($p<0.001$) in IL-13-treated mice (**new Figure 1C** of the revised manuscript). Moreover, quantification of $iNOS^+$ cells demonstrated a significant decrease of M1 macrophages (**new Figure 4B** of the revised manuscript), which is in contrast to the increased numbers of $Ym1^+$ M2 macrophages (**new Figure 4C** of the revised manuscript). As suggested by this Reviewer, we also assessed the M2:M1 ratio in lesions of these mice, which was significantly increased in IL-13-treated mice compared to control mice (**new Figure 4D** of the revised manuscript). Of note, the changed M2:M1 ratio seems to be mainly a result of increased induction of M2 macrophages, as the relative ratio of M1 macrophages to total macrophages did not change. We are enclosing these latter data for the attention of the Reviewer (see graph below – Figure R2).

Thus, IL-13 administration results in decreased total macrophage numbers in lesions, which is paralleled by an absolute and relative increase of M2 macrophages.

Fig R1. Photographs of atherosclerotic lesions of injected *LDLR*$^{-/-}$ mice stained for the presence of total macrophages (A - mac-3), M1 macrophages (B - iNOS) and M2 macrophages (C - Ym-1). Original magnification: 100X and 400X (for the insert).

A

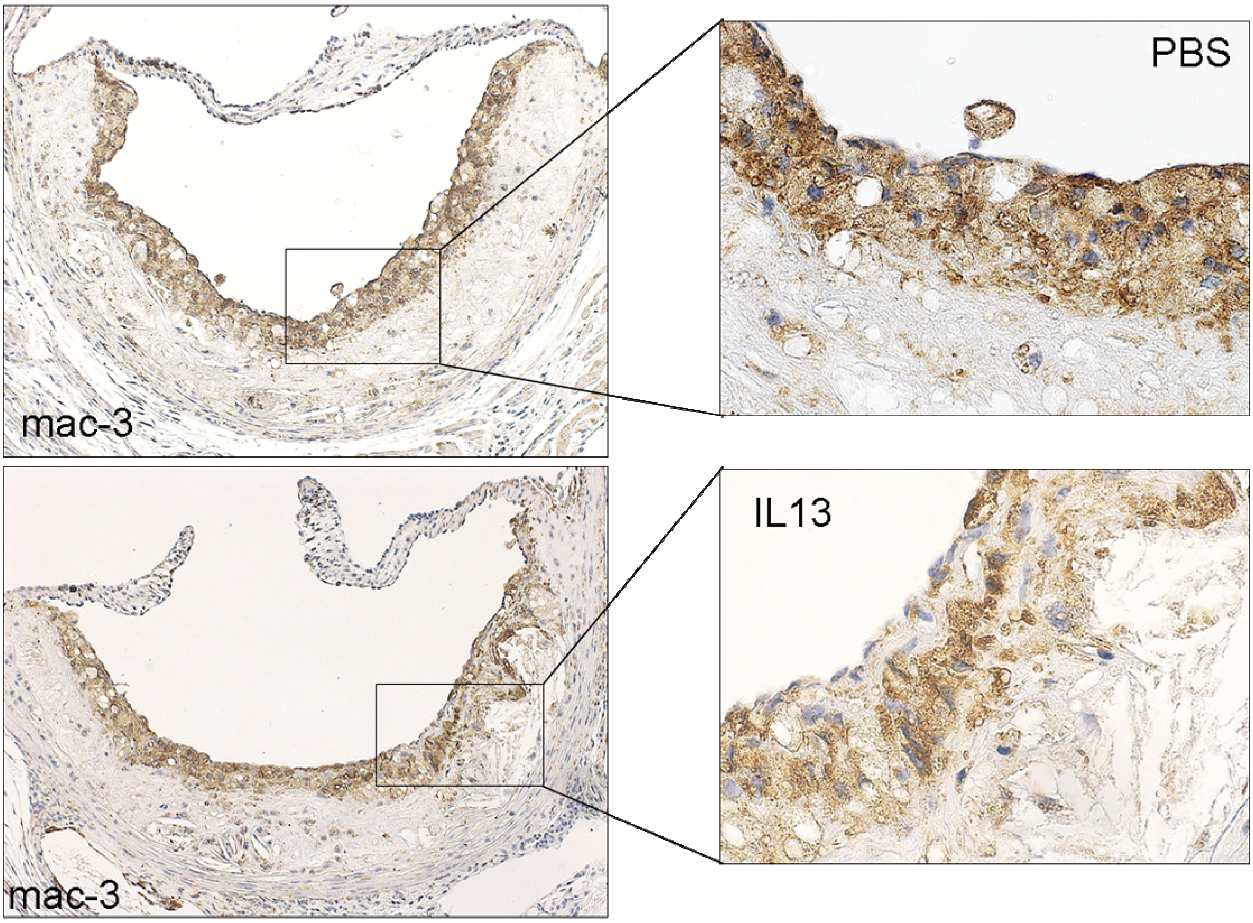

B

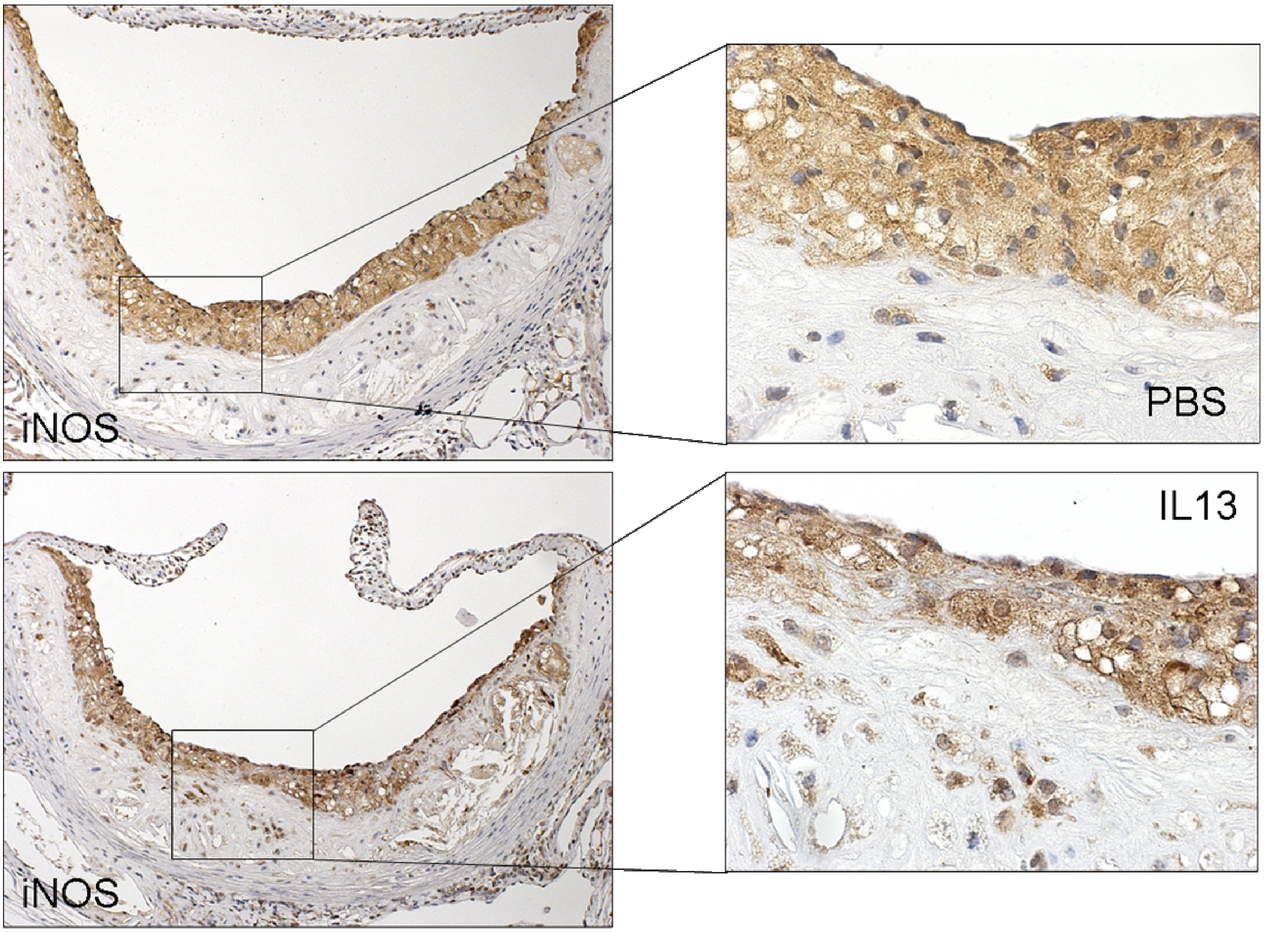

C

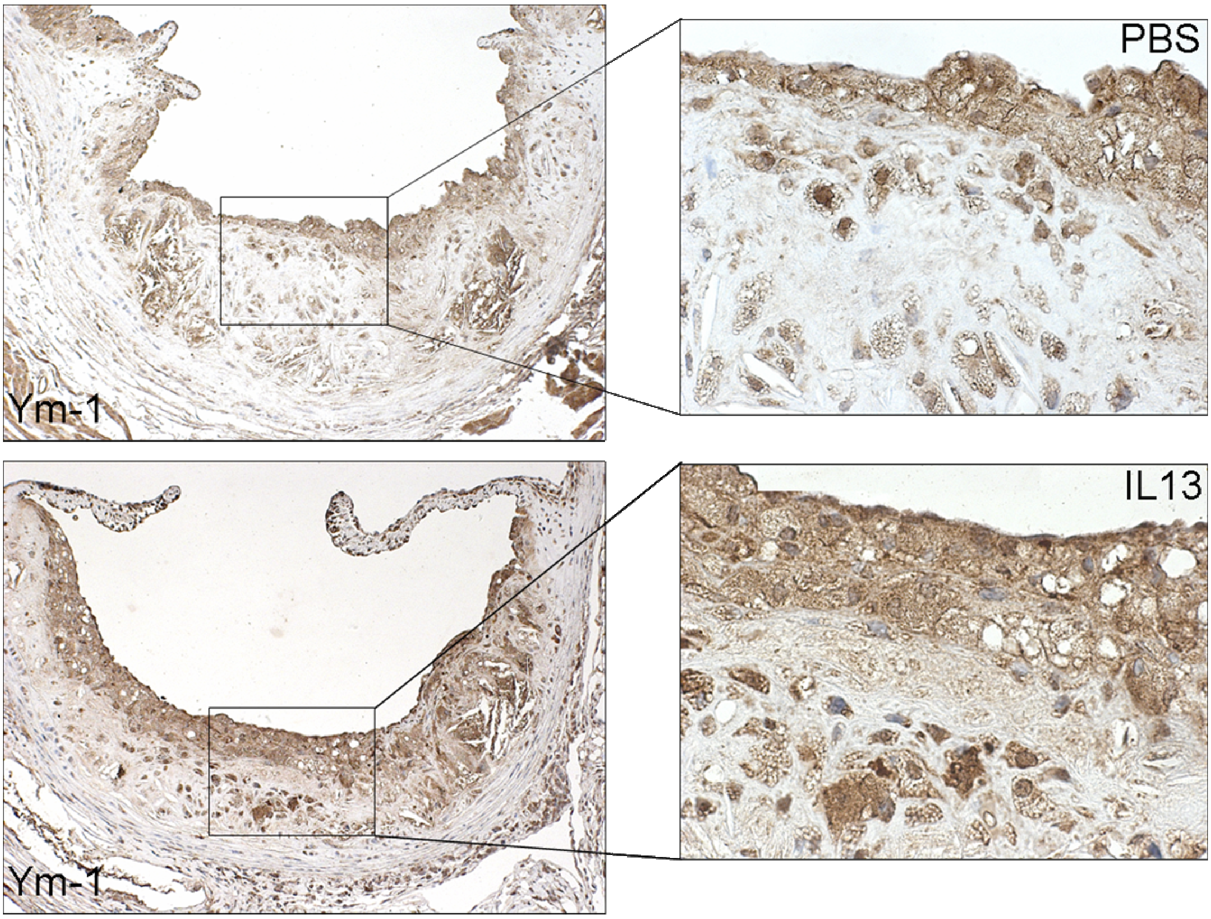

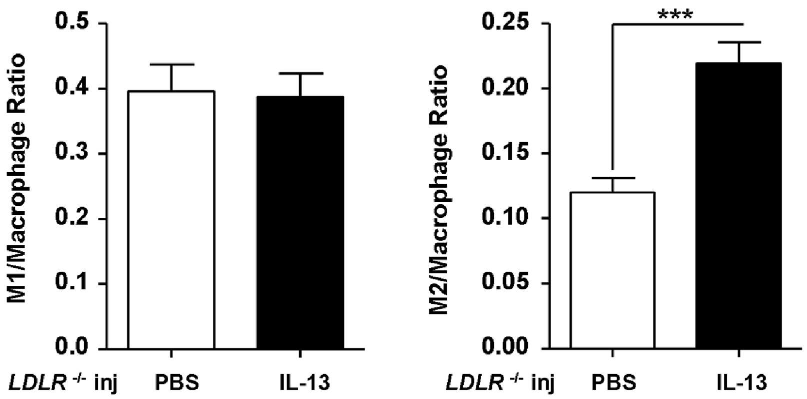

Fig R2. IL-13 administration skews macrophage phenotype towards alternatively activated (M2) macrophages *in vivo*. *LDLR*$^{-/-}$ mice were fed an atherogenic diet for 16 weeks and received bi-weekly intraperitoneal injections with PBS (n=11) or IL-13 (n=13) during the last 5 weeks. The ratio between M1:total macrophages and M2:total macrophages was assessed in lesions of injected *LDLR*$^{-/-}$ mice (Student t-test ***p=0.0001). All data are mean ± SEM values of all mice of each group.

3) In the treatment study major effects on collagen were observed. How was collagen affected in the knockout study?

This is an important suggestion, as it can be expected that recipients of *IL-13*-deficient bone marrow should have less collagen content in their lesions. However, our data identified dramatic differences in lesion size (2-times higher in *IL-13*-deficient mice, **new Figure 6A** of the revised manuscript) and lesion stage, which does not allow direct comparison of qualitative markers, such as collagen, that are known to be influenced by lesion stage. Nevertheless, we have quantified total collagen content in lesions of our bone-marrow transplantation study, and are enclosing these data for the attention of this Reviewer (see graph below – Figure R3). We observed no significant differences between the two groups, but there was a trend towards less collagen content in lesions of *IL-13*-deficient bone-marrow recipients (Student t-test p=0.08). As discussed above the interpretation of these results is virtually impossible given the dramatic differences in lesion size and stage. Therefore, we do not wish to include these data in the manuscript. This would also be the case for total macrophage and iNOS^{+} M1 macrophage areas, which were not different between the two groups. However, we originally decided to include these data only to underscore that there were significant lower numbers of Ym-1^{+} M2 macrophage despite

equal macrophage content. We have included a sentence discussing this issue in the revised version of our manuscript (see page 11, second paragraph).

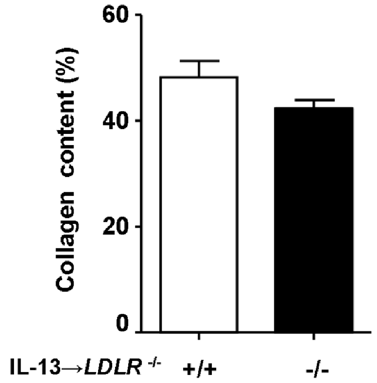

Fig R3. Collagen content in atherosclerotic lesions of *IL-13*-deficient *LDLR*$^{-/-}$ mice. *LDLR*$^{-/-}$ mice were reconstituted with bone marrow from either *IL-13*$^{+/+}$ mice (n=12) or *IL-13*$^{-/-}$ mice (n=14) and fed an atherogenic diet for 16 weeks. Sections were stained with Sirius Red for the presence of collagen, and values represent the percentages of Sirius Red^{+} area/total lesion area. All data are mean ± SEM values of all mice of each group.

4) In figure 1A and figure 5A atherosclerotic lesion size is shown in two different ways. Please use one approach for the aortic root.

As suggested, we are now presenting the **Figure 1A** in the same way as Figure 5A (**now Figure 6A** of the revised manuscript). We feel that readers should be able to appreciate the quantification of the lesions throughout the whole aortic root.

Minor points:

1) In the introduction the paper by King et al. is referred to as a recent paper but it is already 5 years old.

We thank the Reviewer for this comment; this has now been corrected. The sentence now reads *"For example, previous studies reported a pro-atherogenic role for IL-4 (Davenport and Tipping, 2003; King et al., 2002), while a later report found no effect (King et al., 2007)."*

2) The hallmark paper by Bouhlel in Cell Metabolism should be mentioned earlier-on in the paper, in the introduction.

Yes, we agree and have corrected this. This is indeed a very important paper.

3) This reviewer would not call a 5 week period a short treatment, unable to affect plaque size. There is ample literature showing effect of 5 weeks or shorter treatments on atherosclerotic plaque size.

We apologize for being unclear in our statement. We meant to point out that relative to the 16-weeks course of atherogenic diet, 5 weeks of IL-13 administration while maintaining the atherogenic diet is short. We have rephrased this sentence, which now reads: *"As expected with the* <u>*relatively*</u> *short time of administration only during the last 5 weeks* <u>*of a 16-week feeding period*</u>*, there were no differences..."*

4) The title of figure S2 is wrong, stating that there is a phenotypic difference induced by IL-13, but the data in S2 show no difference.

We apologize for mislabeling the title of Figure S2. This has now been corrected**.**

5) The photograph of figure 3b (bottom) should be replaced by a better one. This photograph seems overexposed or with less contrasts then the top one.

As discussed above, we have now added new photographs to this Figure in the revised version of the manuscript (see **Figure 4B**).

Referee #2:

This is an original study that provides novel mechanistic insights into the role of IL-13 in atherosclerosis. The authors used sound murine models of atherosclerosis, and state-of-the-art technology to explore the role of macrophages and T cells. My only concern is that the authors should investigate the role of B cells in more details.

This is an original study that provides novel insights into the role of IL-13 in atherosclerosis. By using different models of atherosclerosis, the authors demonstrated that IL-13-treatment decreased atherosclerosis in apoE-/- mice, whereas transplantation of IL-13 deficient bone marrow to LDLr-/- mice exaggerated atherosclerosis. They also found that IL-13 favoured M1/M2 switch in macrophage phenotype and decreased VCAM-1 dependent monocyte adhesion to the endothelium.

We thank the Referee for her/his kind evaluation and helpful suggestions. As indicated by this Referee, B-cells are important targets for immunological interventions. Therefore, we addressed these aspects in our models, but it seems that IL-13 does not affect B-cell function in the context of the studies carried out for this manuscript.

Comments:

1. page 5. 1st paragraph. What about B cell sub-populations and IgG/IgM antibody levels following IL-13 administration in apoE-/- mice?

We did originally address this specific question in *LDLR*$^{-/-}$ mice that received either IL-13 or PBS, and did not see any differences in antibody levels or lymphocyte populations in the peritoneal cavity (see **Table I** of the revised manuscript). We also had obtained data on splenocytes as well as B-cell subpopulations, which we now included in the revised version of our manuscript. There was no difference in these as well. In addition, as requested by the Reviewer, we now also measured IgM, IgG1, and IgG2c antibody levels as well as splenic B-cell populations in a new set of cholesterol-fed *ApoE*$^{-/-}$ mice that received either IL-13 or PBS (for the egress studies reported in the **new Figure 2** of the revised manuscript). Again, no differences in any of the measured parameters were observed. We are including these data for the kind attention of the Reviewer (see below – Table RI), but given the fact that the data of treated *LDLR*$^{-/-}$ mice are shown in Table I, we feel that it is not necessary to include these data in the revised version of our manuscript.

Table RI

Serum antibodies and splenic population of injected *ApoE*$^{-/-}$ mice.

	**PBS inj → *ApoE*$^{-/-}$ (n=6)**	**IL-13 inj→ *ApoE*$^{-/-}$ (n=7)**
Serum antibody titers		
Total IgM (mg/mL)	0.808 ± 0.15	0.640 ± 0.12
Total IgG1 (mg/mL)	0.391 ± 0.15	0.643 ± 0.24
Total IgG2c (mg/mL)	0.069 ± 0.02	0.096 ± 0.04
Characterization of splenocytes [A]		
$CD43^+$ T cells (% of total)	41.27 ± 2.53	39.15 ± 0.92
$CD220^+$ B cells (% of total)	42.37 ± 2.19	42.02 ± 1.16
$CD43^+IgM^+$ B1 cells (% of Bcells)	7.03 ± 0.33	8.04 ± 0.54
$CD220^+IgM^+$ B2 cells (% of Bcells)	92.32 ± 0.35	91.14 ± 0.56

[A]Cellular sub-populations of splenocytes were analyzed by flow cytometry.

Data are mean ± SEM

2. page 6, 2nd paragraph. How did the authors technically discriminate between GFPhigh and GFPlow monocytes by using intravital microscopy? We anticipate that low GFP levels were barely detectable with this technique.

To discriminate between individual phagocyte subsets, *Cx$_3$cr1*$^{gfp/wt}$*ApoE*$^{-/-}$ mice were injected with a PE-conjugated antibody to Gr-1. Hence, neutrophils appear as PE^+ gfp^-, classical monocytes appear as PE^+gfp^+, and nonclassical monocytes appear as PE^-gfp^+. In the figure below classical monocytes are highlighted by arrows as an example.

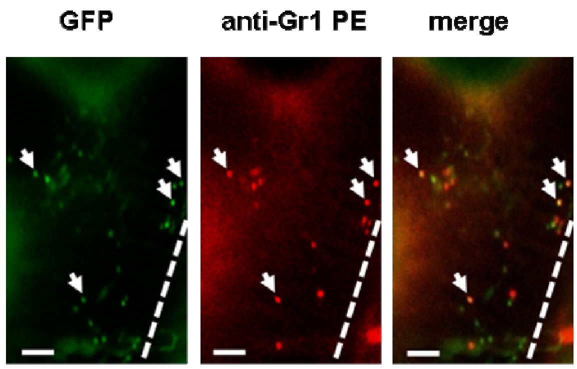

3. page 9. Information about splenic B cell populations in the BMT model would be of interest.

Again, we thank the Reviewer for her/his suggestion. We had also assessed these before and are now including the data in **Supplemental Table SII** of the Support information in the revised version of our manuscript. There was no difference in any of the splenic B-cell populations between the two groups.

Referee #3:

In the current work, Cardilo-Reis et al. evaluate the role of IL-13 in atherosclerosis. IL-13 is an interesting cytokine, given that it polarizes macrophages towards an M2 phenotype, which is considered to be anti-inflammatory and anti-atherogenic. Indeed, lack of IL-13 promotes atherogenesis in LDLR-/- mice, while IL-13 administration in hypercholesterolemic LDLR-/- mice with established lesions modulates the plaque morphology to promote lesion resolution. Therefore, the findings reported herein are highly pertinent to cardiovascular research and of specific interest to the atherosclerosis research community. Importantly, this study points to a novel potential role for IL-13 in the treatment of atherosclerosis, and the key finding is that it may do so in the face of persistent hypercholesterolemia.

Major comments:

1. In the first results section, the authors show a reduction in macrophage recruitment to atherosclerotic lesions, following IL-13 administration.

a. Is IL-13 really modulating recruitment, or could it be promoting egress of macrophages from the lesions?

i. Given the increase in collagen in arterial macrophages, macrophage egress seems probable. This could be addressed using macrophage trafficking techniques such as fluorescently-labeled beads.

We thank the Reviewer for her/his valuable assessment of our manuscript. The question as to why there is a significant decrease in the numbers of macrophages in IL-13 treated mice is of great importance. We had addressed this by looking at the recruitment of monocytes to the carotid arteries of IL-13 treated *ApoE*$^{-/-}$ mice, which indicated decreased recruitment. We agree, that in light of the morphological changes observed that are reminiscent of lesions regression, there is certainly also the possibility of increased macrophage egress as elegantly shown by Fisher and colleagues.

Because CCR7 has been implicated as the key chemokine receptor for mediating macrophage egress during lesion regression, we performed an immunohistochemical evaluation of CCR7 expression in lesions of the cholesterol-fed *LDLR*$^{-/-}$ mice that were treated with IL-13 or PBS, respectively. There was no significant difference in the percentage of CCR7^{+} lesion area between the two groups, suggesting that there is no major role for CCR7-mediated egress. We have now included these data as **Supplemental Figure 3** of the Support information in the revised version of our manuscript.

Importantly, we also performed a new experiment in *ApoE*-deficient mice to assess the possibility of macrophage egress in response to IL-13 administration using fluorescently-labeled beads, as suggested by this Reviewer. *ApoE*-deficient mice were fed an

atherogenic diet for a total period of 6 weeks. After 3 weeks, mice were injected with fluorescent beads to label classical (Ly6C^{hi}) monocytes, which were allowed to be recruited to atherosclerotic lesions within one week. At week 4, one group of mice was sacrificed to obtain baseline levels of fluorescently-labelled lesional macrophages. The other mice received biweekly injections of either IL-13 or PBS for the remaining two weeks, and thereafter were sacrificed to assess the number of remaining labeled macrophages. In this model, macrophage egress is indicated by a significant decrease of beads in atherosclerotic lesions compared to baseline. IL-13 administration had no effect on macrophage egress. However, parallel immunohistochemistry for the macrophage marker mac-2 confirmed significant decreased macrophage content in lesions of IL-13-treated mice compared to PBS-treated mice. Thus, this new experiment confirmed the previously observed decreased macrophage content in response to IL-13 administration, and demonstrated that this effect is independent of macrophage egress. These data are now added as **new Figure 2** in the revised version of our manuscript.

ii. How to explain a reduction in arterial macrophages in response to IL-13 treatment in hypercholesterolemic mice, but comparable arterial macrophage content in lesions of IL-13/LDLR-/- and LDLR-/- mice? Seems inconsistent with a role for IL-13 in macrophage recruitment to the plaque. Wouldn't higher numbers of arterial macrophages in the IL-13/LDLR-/- mice as compared to LDLR-/- mice be expected?

The Reviewer brings up an important question, which we have also addressed in response to question 3 of Reviewer #1. Indeed, one would expect higher numbers of macrophages in the lesions of recipients of *IL-13*-deficient bone marrow. However, as seen in Figure 6, these mice developed more advanced lesion that were two times larger than lesions of control mice. Advanced lesions with increased necrotic areas (as a result of macrophage apoptosis) have typically fewer macrophages. Because lesions were bigger in recipients of *IL-13*$^{-/-}$ bone marrow throughout the entire aortic tree, we do not have the possibility to compare macrophage content of lesions of the same size between these two groups. In our opinion, it is difficult and even misleading to conclude results on macrophage numbers obtained from lesions of different size and stage. We originally included these data only to underscore that there were significant lower numbers of Ym-1^{+} M2 macrophage despite equal macrophage content. We have included a sentence discussing this issue in the revised version of our manuscript (see page 11, second paragraph).

2. In regards to the observed induction of M2 macrophage and concomitant reduction of M1 macrophages, could the authors comment on what they think is happening: are M1 macrophages switching to M2 macrophages (ie regression), or does IL-13 only act on the newly recruited macrophages?

Macrophage plasticity with respect to macrophage polarization is indeed an important question that is topic of ongoing discussions. Data from *in vitro* and *in vivo* experiments suggest that the phenotype of polarized macrophages can be reversed to some extent (Feig et al., 2011; Sica and Mantovani, 2012). In addition, in the microenvironmnet of chronic inflammatory lesions polarizing factors can also act on existing macrophages that are still uncommitted and/or freshly recruited macrophages. For a detailed response, see below.

a. If foam cells (M1) are treated in vitro with IL-13, can they be polarized to an M2 phenotype? Or does IL-13 only have this effect on 'neutral' or 'M0' macrophages?

As suggested by this Reviewer, we now directly tested the possibility if IL-13 has the capacity to revert M1 macrophages towards M2. Thioglycollate-elicited macrophages were either activated with 100 ng/mL IFNγ in the absence or presence of 50 μg/mL CuOx-LDL or left untreated in the absence or presence of CuOx-LDL for 24 hours, and then stimulated with 5 ng/mL IL-13 or medium alone for further 16 hours. Thereafter, expression of M2 markers (Ym-1 and Arg-1) and M1 markers (iNOS and Cxcl10) was assessed by quantitative PCR (see below Table RII). These data demonstrate that IFNγ-activated macrophages as well as CuOx-LDL-loaded macrophages can be fully polarized towards M2, while the M2 polarization of INFγ-activated CuOx-LDL-loaded macrophages (M1 foam cells) by IL-13 appears to be less efficient; i.e. no change in the expression of Ym-1 and iNOS, but induction of Arg-1 and decrease of Cxcl10 expression. Thus, while M1 foam cells still have the potential to respond to IL-13, it is somewhat suppressed; on the other hand M0 foam cells remain fully responsive to IL-13 induced polarization. Interestingly, IFN-γ-activated macrophages that were not loaded with CuOx-LDL also remained fully responsive.

We can only speculate which pathway is more operative in lesions *in vivo*, but given our immunohistochemical findings that indicate an absolute and relative increase only for M2 macrophages (see response to question 2 of Reviewer #1), the polarization of

"uncommitted" macrophages is a possibility. Indeed, our quantitative data on different macrophage populations suggest the existence of macrophages that are neither M1 (iNOS$^+$) nor M2 (Ym-1$^+$). Multiple stages of macrophage activation states exist within atherosclerotic lesions, and the local microenvironment may dictate the activation fate of these macrophages. We believe that most of these macrophages are responsive to IL-13-mediated polarization. We have now added a sentence on this topic in the discussion of the revised version of our manuscript (see page 12, second paragraph), but believe that a more thorough evaluation of this should be part of a separate report.

Table RII

Macrophage polarization towards alternatively activated M2 macrophages *in vitro*.

Gene expression (fold increase over untreated cells)					
		M2 genes		M1 genes	
	Conditions	Ym-1	Arg-1	iNOS	CXCL10
M0	untreated > IL-13	148.9 ± 55.4	59.28 ± 27.1	0.87 ± 0.12	1.08 ± 0.03
M0	untreated > medium	1.03 ± 0.11	1.94 ± 0.63	1.09 ± 0.23	1.08 ± 0.17
M0 foam	CuOx-LDL > IL13	82.22 ± 19.4	33.59 ± 3.96	0.93 ± 0.25	0.66 ± 0.14
M0 foam	CuOx-LDL > medium	1.47 ± 0.38	1.06 ± 0.24	1.42 ± 0.49	1.49 ± 0.43
M1	IFNγ > IL-13	103.4 ± 44.9	57.58 ± 9.83	1.45 ± 0.62	3.20 ± 2.23
M1	IFNγ > medium	1.54 ± 0.24	4.96 ± 1.39	4344 ± 569	47.73 ± 12.3
M1 foam	IFNγ+CuOx-LDL > IL-13	1.36 ± 0.37	10.13 ± 1.99	2416 ± 657	1.88 ± 0.35
M1 foam	IFNγ+CuOx-LDL > medium	1.38 ± 0.21	2.09 ± 0.45	2930 ± 624	6.84 ± 1.66

Thioglycollate-elicited macrophages were activated with either IFNγ in the absence (M1) or presence of CuOx-LDL (M1 foam) or left untreated in the absence (M0) or presence of CuOx-LDL (M0 foam) for 24 hours, and then stimulated with IL-13 or medium alone for further 16 hours. The expression of M2 (Ym-1 and Arg-1) and M1 (iNOS and CXCL10) genes was analyzed by quantitative RT-PCR. Data were normalized to CycB expression and values represent fold increased expression over untreated cells. Data are mean ± SEM of two independent experiments performed in triplicates.

3. In Fig4b, where cholesterol efflux from IFNg- and IL-13-treated macrophages is compared, cholesterol efflux from the two groups likely isn't comparable because the starting specific activity of cholesterol wouldn't be the same in IFN- and IL-13-treated cells, due to the unequal loading of the macrophages from the two groups (ie there is more OxLDL loading in IL-13-primed macrophages compared to IFN-primed macrophages, and consequently the cholesterol label would be more dilute and would likely label a different pool (the lipid droplets) in the IL-13-treated cells as compared to the IFN-treated cells). A better way to directly compare efflux from IFN and IL-13-treated cells would be to first label the macrophages with 3H-cholesterol-OxLDL, to get equal loading, and then assess the effect of IFN and IL-13 treatment on efflux. The treatment of these foam cells would better mimic the situation in vivo, when IL-13 is administered to hypercholesterolemic mice.

We thank the Reviewer for raising a highly relevant point. Indeed, polarization before CuOx-LDL loading may affect equal distribution of the cholesterol label. We now performed a new experiment, in which macrophages were first loaded with CuOx-LDL plus [^{3}H]-cholesterol for 24 hours and then polarized with IFNγ or IL-13 for further 16 hours, respectively. Subsequently, HDL-dependent efflux was measured. Consistent with our previous data, IL-13-activated foam cells displayed a significantly higher efflux capacity compared to IFN-γ-activated foam cells. These data are now included as **new Supplemental Figure S7** of the Support information in the revised manuscript.

4. In Fig 4c, when performing western blots for ABCA1 and ABCG1 expression following OxLDL loading of IFN- and IL-13-treated macrophages, an 'unskewed' or 'neutral' macrophage control (+ or - OxLDL) should be included.

a. Do you expect an intermediate ABCA1 upregulation in 'M0' macrophages as compared to M1 and M2 macrophages, following OxLDL loading?

The Reviewer raises an important point. We did evaluate the expression of both ABC receptors in “neutral” macrophages, which was – as suspected by the Reviewer - intermediate between IFNγ- and IL-13-treated macrophages for ABCA1 and similar to IFNγ-treated macrophages for ABCG1. We are including these data for the kind attention of this Reviewer (see graph below – Figure R4). From unpublished data in our laboratory we became aware that the gene expression profile of “neutral” thioglycollate-elicited macrophages is closer related to the one of alternatively activated macrophages. Therefore, we believe that relating our data to “neutral” macrophages as control is somewhat flawed, and therefore would like to maintain the comparison between the two differentially activated

macrophages in our manuscript. However, in the revised version of our manuscript we now mention also the differences in ABCA1 and G1 expression to “neutral” macrophages. The sentence now reads: *“We therefore investigated the expression levels of the two most important receptors responsible for cholesterol efflux in macrophages, ATP-binding cassette A1 (ABCA1) and G1 (ABCG1) by immunoblotting, and found that upon stimulation with CuOx-LDL the expression of both ABC transporters was significantly up-regulated in foam cells derived from IL-13-activated macrophages compared to IFNγ-activated foam cells (Fig 5C-5E) and non-activated foam cells (data not shown)”.*

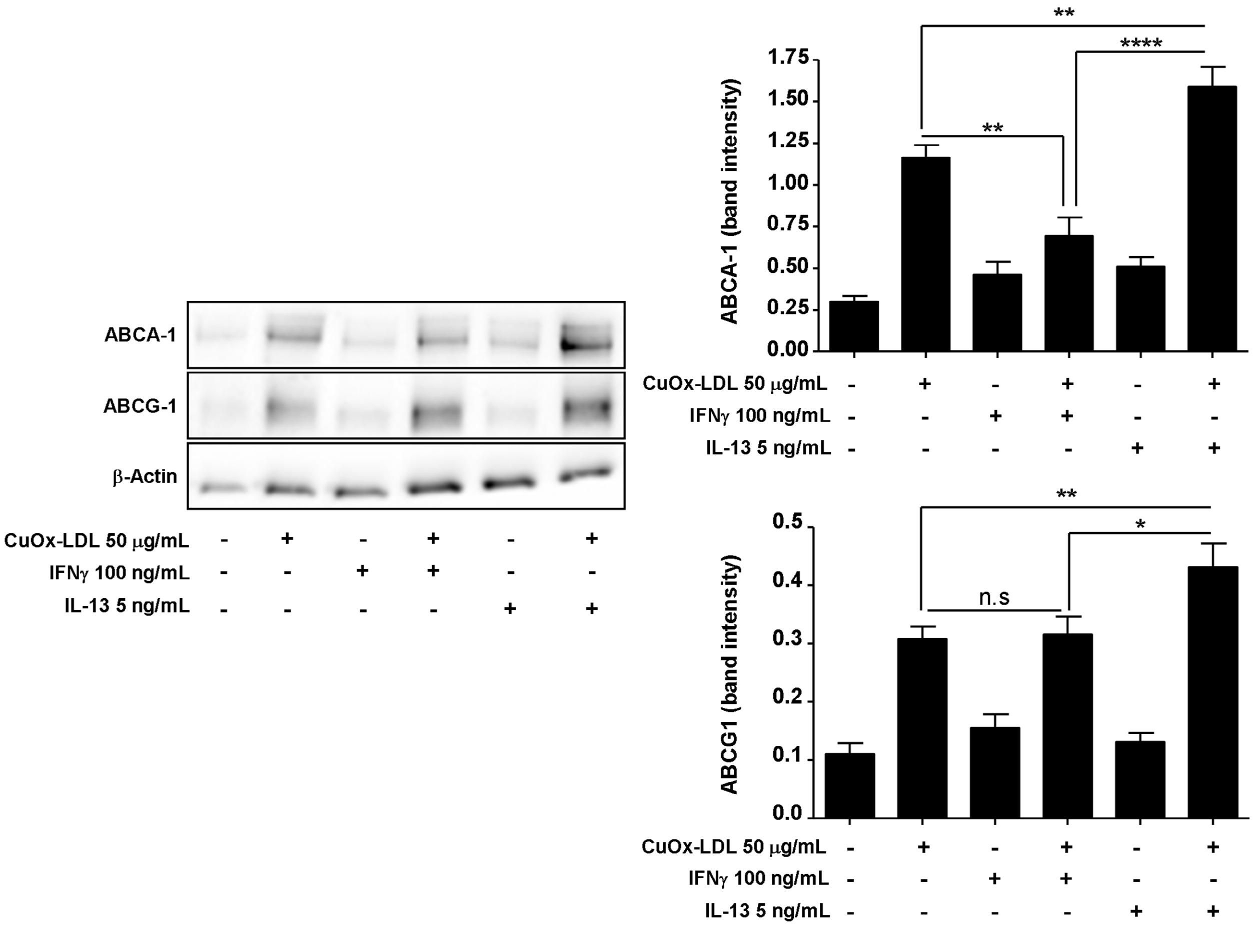

Fig R4. Alternatively activated macrophages (M2) exhibit increased expression of ABC transporters *in vitro*. Thioglycollate-elicited macrophages were left undifferentiated or differentiated with IFNγ or IL-13 into classically (M1) or alternatively (M2), respectively, and then incubated with CuOx-LDL for 24h to generate foam cells. Increased ABCA1 and ABCG1 expression in M2-derived foam cells. Shown is a representative Western blot for the presence of ABCA1, ABCG1, and β-actin in lysates of cells that were treated as indicated. The graphs show the quantification of the band intensity of ABCA1 and ABCG1 related to β-actin (ANOVA *p=0.04, **p=0.0075, ***p=0.0001). All data are mean ± SEM values of three independent experiments performed in triplicates.

b. Can IL-13 rescue the impaired ABCA1 upregulation in IFN-primed M1 macrophages, or does IL-13 further increase ABCA1 expression in macrophages pre-loaded with OxLDL?

In fact, the results of the cholesterol-efflux experiment that was performed in response to Question 3 and is now presented as **Supplemental Figure S7** certainly suggests that IL-13 has the capacity to increase ABCA1 expression in macrophages pre-loaded with CuOx-LDL. We also tried to address the effect of IL-13 to rescue the impaired expression of ABCA1 in IFNγ-primed M1 macrophages by quantitative PCR using the experimental setup described in our response to Question 2a. Unfortunately, under these experimental conditions (i.e. 24 hours preloading with CuOx-LDL in the presence of IFNγ followed by 16 hours stimulation with IL-13 in the absence of additional CuOx-LDL) the mRNA expression of ABCA1 was not informative. Even when "neutral" macrophages pre-loaded with CuOx-LDL were stimulated with IL-13, an effect on ABCA1 expression was not detectable by quantitative PCR. Fully answering this question will require a more thorough evaluation of different experimental conditions, which would take a substantial amount of time, and we believe this should be part of a separate report.

c. Could lesional macrophage ABCA1 expression be assessed in plaques from IL-13 KO and IL-13-treated mice?

Thank you for this good suggestion. We assessed ABCA1 expression in atherosclerotic lesions of *LDLR*$^{-/-}$ mice treated with IL-13 or PBS, respectively. Indeed, consistent with our data, we observed an increase in ABCA1 positive cells in atherosclerotic lesions of IL-13-treated mice compared to controls. We have added these data as **new Figure 5F** in the revised version of the manuscript. For reasons discussed in our response to Question 1a ii, we did not stain lesions of the bone marrow transplantation study.

5. The chosen dose of IL-13 administered was fixed to be 3X that of the IL-13 concentration found in the serum of atherosclerotic mice.

a. How does that compare to IL-13 'basal' levels? After 16 weeks on a hypercholesterolemic diet, are IL-13 serum levels reduced in LDLR-/- mice as compared to prior to commencement of the hypercholesterolemic diet?

We measured serum levels of IL-13 in *LDLR*$^{-/-}$ mice at baseline and after 12 weeks of atherogenic diet. Consistent with an immune activation during atherogenesis in these mice,

we observed a 3-fold increase of serum IL-13 levels from 0.67 ± 0.15 to 2.28 ± 0.23 ng/mL, which we assume are similar to levels observed at 16 weeks of diet. The results are consistent with an endogenous protective response that is induced upon diet-feeding and lesion development. Our data show that exogenous administration of IL-13 can enhance this activity that is seemingly not efficient enough to halt lesion progression. The importance of this endogenous response is underscored by our findings that *IL-13*-deficient *LDLR*$^{-/-}$ mice develop accelerated atherosclerosis. Similar endogenous responses have been described for other anti-atherogenic cytokines such as IL-10, which is also found to increase during lesion progression (Mallat et al., 1999).

Minor comments:

1. Which monocyte populations the Ly6Clo and Ly6Chi cells represent, ie. resident versus inflammatory monocytes, should be specified in the results section to facilitate the reader's interpretation of the results.

We have specified the two monocyte populations as nonclassical Ly6C^{lo} and classical Ly6C^{hi} monocytes in the result section of the revised manuscript.

2. In Fig4A, given that all the 3 doses of HDL tested give the same results, perhaps it is unnecessary to present all 3 - keeping only the 10ug/mL dose, which was used for the efflux in Fig4B, may simplify the message the graph is trying to convey to the reader.

We agree with the Reviewer and have changed the **new Figure 5A** accordingly.

REFERENCES

1. Davenport, P. and Tipping, P.G. (2003) The role of interleukin-4 and interleukin-12 in the progression of atherosclerosis in apolipoprotein E-deficient mice. *Am J Pathol*, 163, 1117-1125.
2. Feig, J.E., Parathath, S., Rong, J.X., Mick, S.L., Vengrenyuk, Y., Grauer, L., Young, S.G. and Fisher, E.A. (2011) Reversal of hyperlipidemia with a genetic switch favorably affects the content and inflammatory state of macrophages in atherosclerotic plaques. *Circulation*, 123, 989-998.
3. King, V.L., Cassis, L.A. and Daugherty, A. (2007) Interleukin-4 does not influence development of hypercholesterolemia or angiotensin II-induced atherosclerotic lesions in mice. *Am J Pathol*, 171, 2040-2047.
4. King, V.L., Szilvassy, S.J. and Daugherty, A. (2002) Interleukin-4 deficiency decreases atherosclerotic lesion formation in a site-specific manner in female LDL receptor-/- mice. *Arterioscler Thromb Vasc Biol*, 22, 456-461.
5. Mallat, Z., Heymes, C., Ohan, J., Faggin, E., Leseche, G. and Tedgui, A. (1999) Expression of interleukin-10 in advanced human atherosclerotic plaques: relation to inducible nitric oxide synthase expression and cell death. *Arterioscler Thromb Vasc Biol*, 19, 611-616.
6. Sica, A. and Mantovani, A. (2012) Macrophage plasticity and polarization: *in vivo* veritas. *J Clin Invest*, 122, 787-795.

2nd Editorial Decision 03 August 2012

Please find enclosed the final reports on your manuscript. We are pleased to inform you that your manuscript is accepted for publication and is now being sent to our publisher to be included in the next available issue of EMBO Molecular Medicine.

If you want to receive an e-mail alert regarding its publication as well as other EMBO Mol Med content, register here:
http://onlinelibrary.wiley.com/getEmailAlert?id=10.1002%2F%28ISSN%291757-4684&originUrl=%2Fjournal%2F10.1002%2F(ISSN)1757-4684%3FglobalMessage=0 .

Our RSS feeds can be found at feed:http://onlinelibrary.wiley.com/rss/journal/10.1002/(ISSN)1757-4684

Please read below for additional IMPORTANT information regarding your article, its publication and the production process.

Congratulations on your interesting work,

Yours sincerely,

Editor
EMBO Molecular Medicine

***** Reviewer's comments *****

Referee #3 (Comments on Novelty/Model System):

The authors now use a variety of different models to test the role of IL-13 in atherosclerosis as well as examining its effects on macrophage populations. Experiments are performed in a well-controlled and proficient manner and the data is presented in a systematic and clear fashion.

Referee #3 (Other Remarks):

The authors have addressed all of the issues raised by the reviewers. In particular, the authors should be commended on their extensive examination of macrophage populations in vivo - which employed many different model systems to address the question of whether or not IL-13 affects macrophages qualitatively, via recruitment or egress as well as quantitatively, by affecting polarization. Their work examining SR expression, LXR expression and the processes of uptake and efflux also greatly contribute to our understanding of these atherogenic and atheroprotective processes in alternatively activated macrophages and foam cells. Overall, this manuscript demonstrates clearly the athero-protective role IL-13 can play via directly polarizing macrophages to a pro-resolving phenotype and points toward an important role as a therapeutic target for treatment of atherosclerosis.
