# Supplementary material for: Interleukin-13 protects from atherosclerosis and modulates plaque composition by skewing the macrophage phenotype
Source: EMBO Mol Med. 2012 Oct 2;4(10):1072–86. doi: 10.1002/emmm.201201374 (PMC3491837; doi:10.1002/emmm.201201374)
Supplement: Supplementary file 2 [file emmm0004-1072-SD2.pdf]

Interleukin-13 protects from atherosclerosis and modulates plaque composition by skewing the macrophage phenotype

Larissa Cardilo-Reis,[1,2] Sabrina Gruber,[1,2] Sabine M. Schreier,[3] Maik Drechsler,[4] Nikolina Papac-Milicevic,[1,2] Christian Weber,[4] Oswald Wagner,[1] Herbert Stangl,[3] Oliver Soehnlein,[4] and Christoph J. Binder[1,2,*]

SUPPORT INFORMATION

TABLE OF CONTENTS:

1. Extended material and methods 3

Animals and intervention studies 3
Evaluation of atherosclerosis 3
Immunohistochemistry and phenotypic analysis of lesions. 4
Serum Total Cholesterol and Triglycerides 4
Lipoprotein isolation and modification 4
Macrophage foam cell assays 5
Immunoblotting 6
Splenocyte cytokine release assay 6
Flow Cytometry 7
Immunoassays 7
Monocyte labeling and macrophage egression assessment 8
Intravital microscopy 8
Statistical analyses 9

2. References 10

3. Supplemental Figures 11

S1: IL-13 administration does not alter cytokine production by splenic T-cells. 11
S2: Morphological analyses of atherosclerotic lesions of *LDLR*$^{-/-}$ mice treated with PBS or IL-13. 12
S3: Equal expression of CCR7 in atherosclerotic lesions of injected *LDLR*$^{-/-}$ mice. 13
S4: Equal expression of CCL2 by endothelial cells of injected *ApoE*$^{-/-}$ mice. 14

S5: IL-13 administration induces alternatively activated M2 macrophages *in vivo*. 15
S6: Alternatively activated macrophages (M2) exhibit increased uptake of CuOx-LDL *in vitro*. 16
S7: Increased HDL-dependent cholesterol efflux by foam cells stimulated with IL-13. 17
S8: Gene expression of alternatively activated M2 macrophages *in vitro*. 18
S9: Successful bone marrow reconstitution in IL-13 chimeric mice. 19
S10: Effect of *IL-13*-deficiency on antibody isotype levels. 20

4. Supplemental Tables 21

Table SI: Overview of experimental data of the bone marrow transplantation study 21
Table SII: Primer sequences 22

EXTENDED MATERIALS AND METHODS

Animals and intervention studies

LDL receptor-deficient mice (*LDLR*$^{-/-}$) and C57BL/6J were from The Jackson Laboratories (Bar Harbor, Maine, USA); *IL-13*$^{-/-}$ mice were a kind gift of Dr. Thomas Wynn (NIAID/NIH, Bethesda, USA). All mice were on a C57BL/6J background and were bred in-house. All experimental protocols were approved by the institutional animal experimentation committee and the Austrian Ministry of Science.

Bone marrow transplantation (BMT) studies were performed as previously described (Binder et al, 2004). Thirty 8-week-old male *LDLR*$^{-/-}$ mice were given a single dose of 9-Gy lethal total body-irradiation to eliminate endogenous bone marrow stem cells, and irradiated mice were injected intravenously with $2x10^6$ bone marrow cells harvested from either *IL-13*$^{-/-}$ (n=15) or *IL-13*$^{+/+}$ (n=15) mice. Mice were fed regular chow diet for 4 weeks after BMT to allow for bone marrow reconstitution and then switched to an atherogenic diet containing 21% fat and 0.2% cholesterol (TD88137, Ssniff Spezialdiäten GmbH, Soest, Germany) for an additional 16 weeks to induce lesion formation. Three mice (one in the *IL-13*$^{-/-}$ and two in control group) were *a priori* excluded from final analyses because they developed skin lesions and lost weight, or due to technical problems of tissue collection. One mouse from the control group was excluded as statistical outlier, which however did not alter the statistical significance of the results.

For the atherosclerosis intervention study, twenty-four 12 week-old female *LDLR*$^{-/-}$ mice were fed an atherogenic diet (Ssniff) for a total of 16 weeks to induce lesion formation. At week 11, mice were divided randomly into two groups and injected intraperitoneally with PBS (n=11) or IL-13 (50 ng/mouse R&D systems, Minneapolis, Minnesota, USA; n=13) twice per week for the following remaining 5 weeks. For the isolation of RNA from atherosclerotic lesions present in the aortic arch and valves, sixteen 12 week-old female *LDLR*$^{-/-}$ mice were fed an atherogenic diet (Ssniff) for a total of 12 weeks to induce lesion formation. At week 8, mice were randomly divided into two groups and injected intraperitoneally with PBS (n=7) or IL-13 (50 ng/mouse R&D systems, n= 9) twice per week for the following remaining 4 weeks.

Evaluation of atherosclerosis

The extent of atherosclerosis was determined in a blinded fashion in *en face* preparations of the entire aorta, as well as in cross sections through the aortic origin, by computer-assisted image analysis as previously described (Binder et al, 2004). At time of sacrifice, animals were perfused with PBS, followed by formalin (4% paraformaldehyde in PBS, pH 7.4), and the entire mouse aorta was dissected from the proximal ascending aorta to the bifurcation of the iliac artery. Adventitial fat was removed, and the whole aorta was opened longitudinally, pinned, stained with Sudan IV, and photographed with a digital camera. Total aortic area and aortic lesion area were calculated using Adobe Photoshop CS3 (Adobe Systems, San Jose, California, USA) and ImageJ 1.41 software. Results are reported as percentage of lesion area per total aortic surface area. As a second assessment of atherosclerosis, cross-sections of the aortic origin were quantified for lesion size and phenotype. Serial sections (5 µm in thickness) were cut through a 400 µm segment of the aortic root, starting with the appearance of all 3 valve leaflets. For each mouse, 8 sections separated by 50 µm were examined. Each section was stained with H&E and photographed using the AxioVison software (Carl Zeiss AG, Jena,

Germany). Total lesion area was quantified using Adobe Photoshop CS3 and ImageJ 1.41 softwares.

Immunohistochemistry and phenotypic analysis of lesions.

Lesion phenotype was determined by the content of collagen, size of necrotic core area, and the presence of macrophages, smooth-muscle cells, T-cells, classical activated (M1) macrophages, alternatively activated (M2) macrophages, ABCA1 expressing cells, and CCR7 expression in lesions of equal size. For the collagen content, sections were stained with Sirius Red, and for the assessment of necrotic cores, sections were stained with a modified elastic-trichrome stain. The photographed images were analyzed using ImageJ 1.41 software to determine the percentage of collagen and necrotic core area, respectively. For the presence of macrophages, smooth-muscle cells, T-cells, M1 macrophages and M2 macrophages immunohistochemistry was performed using antibodies against mouse Mac-3 (1:50 rat anti-mouse, clone M3/84, BD-Biosciences Pharmingen, San Diego, California, USA), smooth-muscle cell actin (1:10,000 polyclonal rabbit anti-mouse, Sigma-Aldrich), CD3 (1:800 polyclonal rabbit anti-human/mouse, DAKO, Glostrup, Denmark), iNOS (1:500 polyclonal rabbit anti-human/rat/mouse, ABCAM, Cambridge, UK), CD206 (1:20 rat anti-mouse, clone MR5D3, BioLegend, San Diego, California, USA) and Ym1/2 (1:1,000 polyclonal rabbit anti-mouse, a kind gift of Dr. Shioko Kimura, NIH/NCI, Bethesda, USA). For the evaluation of CCR7 and ABCA1 expression, immunohistochemistry was performed using antibodies against mouse CCR7 (1:700 monoclonal rabbit anti-human/rat/mouse, clone Y59, ABCAM) and mouse ABCA1 (1:1,000 polyclonal rabbit anti-mouse, Novus Biological, Littleton, Colorado, USA). Briefly, sections were incubated with the primary antibody overnight at 4°C, blocked for endogenous peroxidase with 3%H_2O_2 and following several washes incubated with a secondary biotinylated anti-rat (1:100 rabbit anti-rat IgG, DAKO) or anti-rabbit (1:500 goat anti-rabbit IgG, Vector Laboratories, Burlingame, California, USA) antibody for 30 min at room temperature. Then, sections were incubated with streptavidin-HRP Ultrasensitive (1:1,000 Sigma-Aldrich, St. Louis, Missouri, USA) for 30 min at room temperature in the dark, and subsequently developed with DAB (Liquid DAB + Substrate chromogen System, DAKO). The sections were photographed and the stained area was quantified using ImageJ 1.41 software or positive cells were counted.

Serum Total Cholesterol and Triglycerides

At time of sacrifice, blood samples were collected from the vena cava of all mice. Total serum cholesterol and triglycerides were measured by enzymatic methods using an automated analyzer AU5400 – Chemistry System (Beckman Coulter, Brea, California, USA).

Lipoprotein isolation and modification

Human LDL and HDL were isolated from EDTA-plasma of healthy donors after overnight fasting by differential density ultracentrifugation on OTD Combi (Sorvall, Thermo Fisher Scientific, Waltham, Massachusetts, USA) over the density range of d 1.019 to 1.063 and 1.21 g/mL, respectively as described (Binder et al, 2003). The quality of LDL-preparations was checked by lipoprotein electrophoresis. LDL was sterile filtered and stored at 4°C. CuOx-LDL and MDA-LDL were prepared as described previously (Binder et al, 2003). Protein

concentrations were determined by Lowry or BCA-method according to manufacturer's instructions (Pierce, Thermo Fisher Scientific). All lipoprotein preparations used for cell culture were tested for endotoxin levels by chromogenic Limulus amoebocyte assay (QCL-1000; BioWhittaker Inc, Wakersville, Maryland, USA) and contained less than 1 ng lipopolysaccharides/mg protein.

Macrophage foam cell assays

Isolation of peritoneal macrophages. For all *in vitro* experiments, C57BL/6J mice, 12-16 weeks of age, were injected intraperitoneally with 2 mL of 3% thioglycollate (Difco, Thermo Fischer Scientific). After 3 days, thioglycollate-elicited macrophages were harvested and plated in full culture medium (RPMI 1640 media, 100 U/mL penicillin, 100 µg/mL streptomycin and 50 µg/mL gentamicin; all Invitrogen, Carlsbad, California, USA) containing 10% heat-inactivated fetal calf serum (FCS) at a density of $3x10^5$ cells/well in 48-well plate; $5x10^5$ cells/well in 24-well plate; $1.5x10^6$ cells/well in 12-well plate or $3.5x10^6$ cells/well in 6-well plate for lipid uptake, cholesterol efflux, RNA/protein isolation and cellular cholesterol quantification experiments, respectively.

Macrophage differentiation and generation of foam cells. Macrophages were differentiated into classical activated macrophages (M1) with 100 ng/mL IFNγ (R&D systems) or alternatively activated macrophages (M2) with 5 ng/mL IL-13 (R&D systems) in full culture medium containing 10% FCS for 16 hours at 37°C. Following differentiation, cells were washed once with PBS and stimulated with 50 µg/mL CuOx-LDL in the presence or absence of HDL 10µg/mL in full culture medium containing 1% mouse serum for 24 hours at 37°C.

Lipid uptake – Oil Red O staining. After foam cell generation, cells were washed once with PBS and fixed with a solution of formalin-sucrose (4% paraformaldehyde, 4% sucrose in PBS) for 20 min at room temperature. Following a wash with 60% isopropanol, cells were stained with Oil Red O (Sigma-Aldrich) diluted 6:4 parts in dH_2O for 20-30 min at room temperature. After washing several times with dH_2O, 3 random-fields were counted for positive and negative stained cells under the microscope. The data are expressed as percentage of Oil Red O positive cells per total cells

Cellular cholesterol content. Foam cells were lysed with a solution of 0.1M NaOH, and cellular lipids were extracted by hexane/isopropanol (3:2). Total cholesterol content was determined using the Amplex Red Cholesterol Assay kit (Invitrogen). Values are related to total protein content of each sample.

Cholesterol efflux assay. Following macrophage differentiation, cells were stimulated with 50 µg/mL CuOx-LDL plus 1 µCi [H^3]-cholesterol (Perkin Elmer, Waltham, Massachusetts, USA) in full culture medium for 24 hours at 37°C (Fig 5B) or macrophages were loaded with 50 µg/mL CuOx-LDL plus 1 µCi [H^3]-cholesterol in full culture medium for 24 hours at 37°C prior to macrophage differentiation with either 100 ng/mL IFNγ or 5 ng/mL IL-13, respectively in full culture medium for further 16 hours (Fig S7 of Support information) . Thereafter, cells were equilibrated in serum free-full culture medium for 2h at 37°C. After equilibration, cells were incubated with or without 10 µg/mL HDL plus 50 µg/mL CuOx-LDL in serum free-full culture medium for 6 hours at 37°C. Supernatants were collected and spun down (at 3,000 rpm for 10 min) to remove cellular debris, and the radioactivity in the supernatants was measured using a liquid scintillation counter (β-counter 2000 CA; TRI-CARB Liquid Scintillation Analyzer, Packard). Cells were lysed in 0.1M

NaOH and the cell-associated radioactivity was measured as described above. All measured counts were corrected for total cellular protein content as determined by the Bradford method (Bio-Rad Protein Assay, Bradford Laboratories, Hercules, California, USA). The percent efflux was calculated as (media dpm)/(cell+media dpm) x 100 and the HDL dependent-efflux was calculated as (mean % efflux with HDL)-(mean % efflux w/o HDL).

RNA/protein isolation. Following foam cell generation, total RNA was isolated using the RNeasy Mini Kit (PeqLab, Vienna, Austria) and 500 ng of total RNA was reverse-transcribed using the High Capacity cDNA Reverse Transcription kit (Applied Biosystems, Carlsbad, California, USA). Quantitative real-time PCR was performed using iTaq SYBR Green Supermix with ROX Dye (Bio-Rad Laboratories, Hercules, California, USA) in a CFX96 Real-time System (Bio-Rad Laboratories). All data were normalized to Cyclin B (CycB) and expressed as fold change over control (untreated cells). Primer sequences are available in the Table SII. For protein detection, cells were incubated for 10 min at room temperature with a lysis buffer containing 62.5mM Tris, 8M Urea, 20mM EDTA, 20mM EGTA, 10% glycerol, 1% SDS (pH6.8), and lysates were sonicated for 2 seconds and stored at -20°C for further analyses.

Immunoblotting

Samples were separated by SDS-PAGE and blotted on nitrocellulose-membranes (Trans-Blot® Transfer Medium, Bio-Rad Laboratories). Membranes were blocked in 5% non-fat dry milk in TBS containing 0.1% Tween 20 for 60 min at room temperature. Following 3 washing steps, blots were probed using anti-ABCA1 (rabbit IgG anti-mouse, 1:500, Novus Biological) or anti-ABCG-1 (rabbit IgG anti-mouse, 1:2,000, Novus Biological) or anti-β-actin (mouse IgG2a anti-mouse, 1:5,000, Sigma-Aldrich) antibodies overnight in washing buffer (TBS + 0.1% Tween 20) containing 3 % BSA at 4 °C. After 3 washing steps, blots were incubated with a secondary HRP-labeled goat anti-rabbit (1:10,000, Sigma-Aldrich) or goat anti-mouse IgG (1:10,000, Bio-Rad Laboratories) in washing buffer containing 5% non-fat dry milk for 60 min at room temperature. After further washing, membranes were developed using a 1:1 solution of Luminol/Enhancer and Stable Peroxidase (Super Signal® West Dura Extended Duration Chemiluminescent Substrate, Pierce Biotechnology, Rockford, Illinois, USA), and chemiluminescence was detected using a CCD camera (FUSION FX7, PEQLAB Biotechnologie GmbH, Erlangen, Germany). AlphaEase FC software (Alpha Innotech Corporation, San Leandro, California, USA) was used for quantification.

Splenocyte cytokine release assay

Total Splenocytes (0.25×10^5) were seeded into 96-well round-bottom plates in full culture medium containing 10% FCS in a final volume of 100 µl. Quadruplicate cultures of splenocytes from individual mice were incubated for 72 hours at 37°C/5% CO_2 either alone or in the presence of 10 µg/mL plate-bound anti-mouse CD3 (clone 145-2C11, BD Biosciences — Pharmingen) and 10 µg/mL soluble anti-mouse CD28 (clone 37.51, BD Biosciences — Pharmingen) to induce maximal activation of T-cells. The amounts of INF-γ, IL-4, IL-5, IL-10 and IL-13 were determined by a fluorescent bead immunoassay in undiluted supernatants (FlowCytomix 5-plex kit, eBioscience, Vienna, Austria).

Flow Cytometry

At time of sacrifice, peritoneal exudate cells (PEC) were harvested by peritoneal lavage using ice-cold HBSS supplemented with 2% FCS. Total white blood cells were isolated from blood collected in EDTA-tubes via the vena cava. Blood was diluted 1:1 in a solution of PBS containing 2% Dextran (Sigma-Aldrich) and incubated for 40 min at 37°C to separate the red blood cells. All cells were washed and incubated with an anti-Fcγ receptor blocking antibody (anti-mouse CD16/CD32, clone 93, 1:200, eBioscience) for 30 min at 4°C. T-cells, B-cells and macrophages in PEC were assessed using FITC-labeled anti-CD23 (clone B3B4, 1:800, BD Biosciences – Pharmingen), phycoerythrin (PE)-labeled anti-CD5 (clone 53-7.3, 1:100, eBioscience), PercP-labeled anti-CD19 (clone 1D3, 1:200, BD Biosciences – Pharmingen), and APC-labeled anti-CD11b/Mac-1 (clone M1/70, 1:800, eBioscience); macrophage subpopulations were determined using FITC-labeled anti-CD206 (clone MR5D3, 1:200, BioLegend), PE-labeled anti-CD80 (clone 16-10A1, 1:200, BD Bioscience – Pharmingen) and APC-labeled anti-F4/80 (clone BM8, 1:200, Invitrogen). Peripheral blood cells were analysed for the presence of resident monocytes, inflammatory monocytes, neutrophils and B cells using FITC-labeled anti-Ly6C (clone HK1.4, 1:200, BioLegend), PE-labeled anti-Ly6G (clone 1A8, 1:2000, BioLegend), PercP-labeled anti-CD19 (clone 1D3, 1:200, BD Biosciences – Pharmingen) and APC-labeled anti-CD11b/Mac-1 (clone M1/70, 1:800, eBioscience). All stains were performed in 100 µL of FACS buffer (PBS + 2% FCS) for 30 min at 4°C in darkness, followed by two washes. Stained cell populations were analyzed by multiparameter flow cytometry using a BD FACSCalibur (BD Bioscience, Franklin Lakes, New Jersey, USA). More than 10^5 cells were acquired per sample, with dead cells excluded by forward and side scatter properties and data were analyzed using FlowJo 7.6.4 software (Tree Star, Inc., Oregon Corporation, Ashland, Oregon, USA).

Immunoassays

Antibody titers to MDA-LDL were determined by chemiluminescent ELISA as previously described (Binder et al, 2003). Total isotype levels were determined by a chemiluminescent-based sandwich ELISA using matched pairs of specific antibodies for capture and detection, followed by incubation steps with AP-conjugated NeutrAvidin (1:10,000 in TBS-BSA, Perkin Elmer) at room temperature and Lumiphos Plus (P-701, 30% solution in water, Lumigen, South Field, Michigan, USA). Chemiluminescence was detected using a luminescence reader (BioTek Synergy 2, BioTek, Winooski, Vermont, USA). The following antibodies were paired with appropriated AP-conjugated or biotinylated detecting antibodies, respectively: anti-mouse IgM (coated at 2 µg/mL, Sigma-Aldrich) and AP-conjugated goat anti-mouse IgM (1:20,000 in TBS-BSA, Sigma-Aldrich), anti-mouse IgG1 (coated at 2 µg/mL, BioLegend) and biotinylated anti-mouse IgG1 (at 0.5 µg/mL in TBS-BSA, BD Bioscience), anti-mouse IgG2c (coated at 1 µg/mL, AbDSerotec, Oxford, United Kingdom) and biotinylated goat anti-mouse IgG2c (1:4,000 in TBS-BSA, Jackson ImmunoResearch Laboratories, West Grove, Pennsylvania, USA). Isotype levels were measured at following three serum dilutions: 1:10,000, 1:30,000 and 1:90,000 for IgM, 1:50,000, 1:150,000, 1:450,000 for IgG1 and 1:250,000, 1:500,000, 1:1,000,000 for IgG2c. Results were calculated on a standard curve, which was generated using respective purified isotypes obtained from Biolegend (IgM and IgG1) or Southern Biotech (IgG2c, SouthernBiotech, Birmingham, Alabama, USA).

Monocyte labeling and macrophage egression assessment

Macrophage emigration from atherosclerotic lesions were analyzed by a fluorescent labelled-bead tracking technique previously described (Potteaux et al, 2011). Twenty-two male, 8 weeks old, *ApoE* $^{-/-}$ mice (C57BL/6J background) were fed a standard western diet containing 21% fat and 0.2% cholesterol (Altromin Spezialfutter GmbH & Co.KG, Lage, Germany) for 6 weeks to induce lesion formation. At week 3, circulating classical Ly6C^{hi} monocytes were labeled by intravenous (i.v) injection of 1 μm Fluoresbrite green fluorescent (YG) plain microspheres (Polysciences Inc., Warrington, Pennsylvania, USA) diluted 1:4 in sterile PBS, 24 hours after an i.v. injection of 250 μl clodronate-loaded liposomes to deplete monocytes. Mice were not manipulated for the following week to allow latex-beads^{+}monocytes to accumulate within atherosclerotic plaques. At week 4, mice were divided into three groups randomly. One group was sacrificed for the quantification of bead and macrophage (mac-2^{+}cells) content (baseline, n=7); the remaining two groups received biweekly intraperitoneal injections of either PBS (n=7) or IL-13 (50 ng/mouse, R&D systems n=8) for the remaining 2 weeks. All animal experiments were approved by the local ethical committee (Regierung von Oberbayern). At the end of week 6, numbers of beads per plaque area in the aortic root and mac-2^{+}cells per total DAPI^{+}cells were quantified in cross-sections of atherosclerotic lesions of PBS- and IL-13-treated mice. Macrophage egress was assessed by the number of beads present within the plaque area at the end of the experimental approach compared to baseline (week 4).

Intravital microscopy

Leukocyte endothelial interactions and expression of endothelial adhesion molecules were analyzed by intravital microscopy of the left carotid artery. Sixteen male, 8 weeks old, *Cx₃cr1*$^{gfp/wt}$ *ApoE* $^{-/-}$ mice (C57BL/6J background, for leukocyte endothelial interactions) and sixteen male, 8 weeks old, *ApoE* $^{-/-}$ mice (C57BL/6J background, for endothelial adhesion molecules expression) were fed a standard western diet containing 21% fat and 0.2% cholesterol (Altromin) for 6 weeks to induce lesion formation. At week 4, they were divided into two groups randomly and injected intraperitoneally with PBS (n=8) or IL-13 (50 ng/mouse, R&D systems n=8) twice per week for the remaining 2 weeks. At the end of this period, mice were anaesthetized with ketamine/xylazine and the left carotid artery was exposed as described previously (Drechsler et al, 2010). All animal experiments were approved by the local ethical committee (Regierung von Oberbayern). Circulating leukocytes were labeled by injection of a PE-labeled anti-mouse Gr1 antibody (clone RB6-8C5, 5μg, eBioscience) via an intravenous catheder 5 min prior to recording. Intravital microscopy was performed using an Olympus BX51 microscope (Olympus, Hamburg, Germany) equipped with a beam splitter to enable synchronized dual-channel recording, a Hamamatsu 9100-02 EMCCD camera (Hamamatsu Photonics, Hamamatsu City, Japan), and a 10x saline-immersion objective. For image acquisition and analysis Olympus Cell^{r} software was used. Adherent monocytes (GFP) and neutrophils (GFP^{-}/PE^{+}) were recorded at the bifurcation. For luminal detection of VCAM-1 or CCL-2 presented on the endothelium, 50 μl of Protein G Fluoresbrite YG Micropheres (Polysciences Inc) were coupled to 50 μg of polyclonal antibodies against mouse VCAM-1 (eBioscience) or mouse CCL2 (eBioscience) as described recently (Engel et al, 2011). Antibody/bead complexes were injected intravenous and allowed to circulate for 15 min. Complexes immobilized along the carotid artery were detected by intravital microscopy as described above.

Statistical analysis

Statistical analyses were performed using GraphPadPrism 4.03 software (GraphPad Software, Inc., La Jolla, California, USA). Results were analyzed by one-way analysis of variance (ANOVA) with the post-test Bonferroni's multiple comparison tests for all the *in vitro* data. Student's unpaired *t*-test was used for results of *in vivo* studies (unless indicated differentially). Data are presented as mean ± SEM and $P < 0.05$ was considered significant.

REFERENCES

Binder CJ, Hartvigsen K, Chang MK, Miller M, Broide D, Palinski W, Curtiss LK, Corr M, Witztum JL (2004) IL-5 links adaptive and natural immunity specific for epitopes of oxidized LDL and protects from atherosclerosis. *J Clin Invest* **114**(3)**:** 427-437

Binder CJ, Horkko S, Dewan A, Chang MK, Kieu EP, Goodyear CS, Shaw PX, Palinski W, Witztum JL, Silverman GJ (2003) Pneumococcal vaccination decreases atherosclerotic lesion formation: molecular mimicry between Streptococcus pneumoniae and oxidized LDL. *Nat Med* **9**(6)**:** 736-743

Drechsler M, Megens RT, van Zandvoort M, Weber C, Soehnlein O (2010) Hyperlipidemia-triggered neutrophilia promotes early atherosclerosis. *Circulation* **122**(18)**:** 1837-1845

Engel D, Beckers L, Wijnands E, Seijkens T, Lievens D, Drechsler M, Gerdes N, Soehnlein O, Daemen MJ, Stan RV, Biessen EA, Lutgens E (2011) Caveolin-1 deficiency decreases atherosclerosis by hampering leukocyte influx into the arterial wall and generating a regulatory T-cell response. *FASEB J* **25**(11)**:** 3838-3848

Potteaux S, Gautier EL, Hutchison SB, van Rooijen N, Rader DJ, Thomas MJ, Sorci-Thomas MG, Randolph GJ (2011) Suppressed monocyte recruitment drives macrophage removal from atherosclerotic plaques of Apoe-/- mice during disease regression. *J Clin Invest* **121**(5)**:** 2025-2036

SUPPLEMENTAL FIGURES

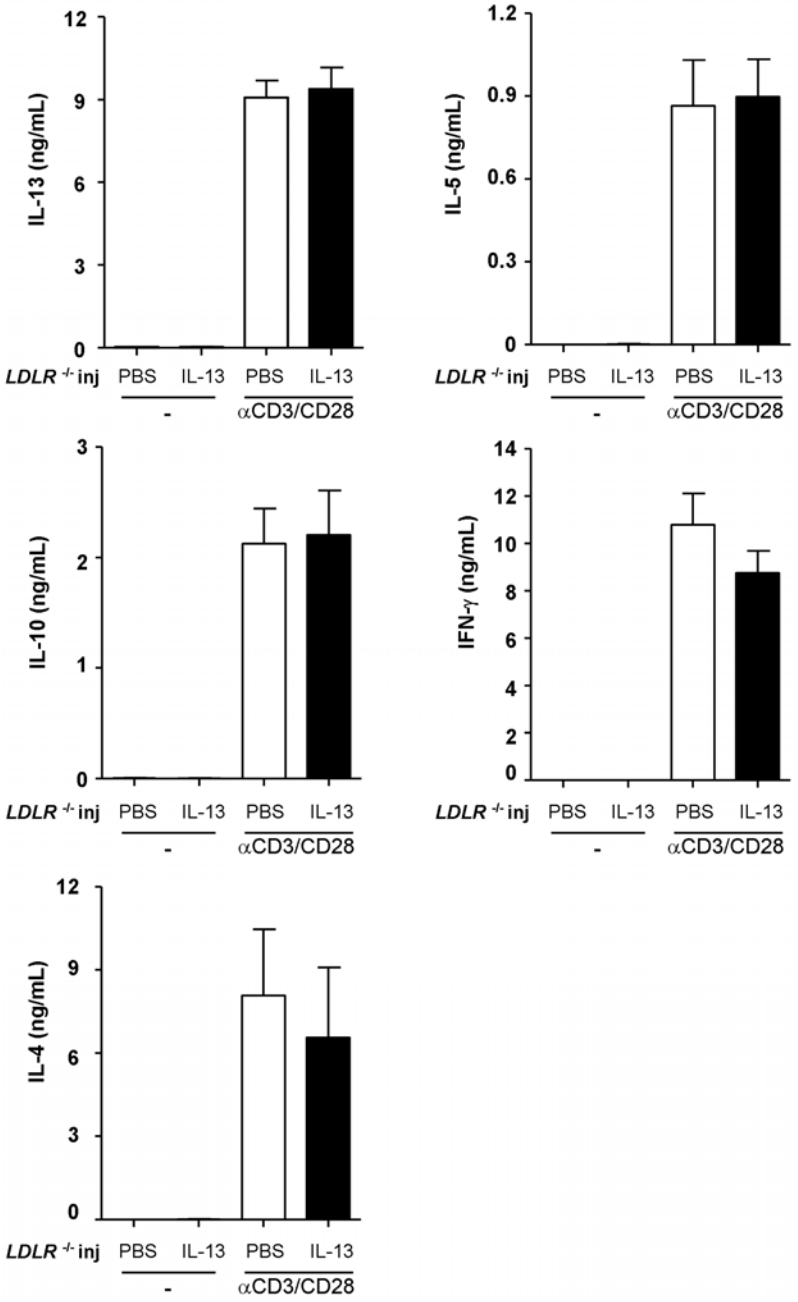

Fig S1. IL-13 administration does not alter cytokine production by splenic T-cells. *LDLR*$^{-/-}$ mice were fed an atherogenic diet for 16 weeks and received biweekly intraperitoneal injections with PBS (n=11) or IL-13 (n=13) during the last 5 weeks. At time of sacrifice spleens were collected from all mice and splenocytes stimulated with or without anti-CD3/CD28 *in vitro*. *LDLR*$^{-/-}$ mice from both groups show similar production of Th2 (IL-13, IL-5, IL-4 and IL-10) and Th1 (IFN-γ) cytokines. Data are presented as mean ± SEM ng/mL of individual cytokines in splenocyte cultures of all mice of each group.

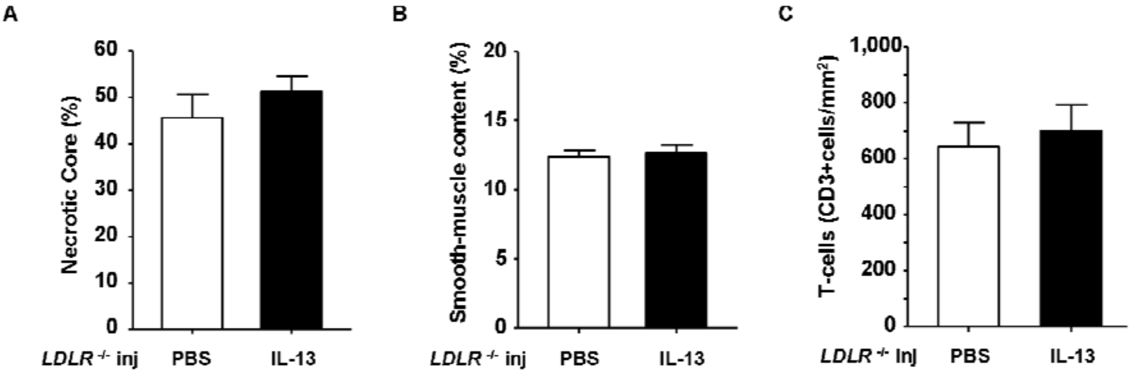

Fig S2. Morphological analyses of atherosclerotic lesions of *LDLR*$^{-/-}$ mice treated with PBS or IL-13. *LDLR*$^{-/-}$ mice were fed an atherogenic diet for 16 weeks and received biweekly intraperitoneal injections with PBS (n=11) or IL-13 (n=13) during the last 5 weeks. **(A)** Equal necrotic core area in lesions of injected *LDLR*$^{-/-}$ mice. Values represent percentages of necrotic core area/total lesion area. **(B)** Equal smooth-muscle cell content in lesions of injected *LDLR*$^{-/-}$ mice. Sections were stained with an anti-smooth-muscle cells actin specific antibody, and values represent the percentages of smooth-muscle actin^{+} area/total lesion area. **(C)** Equal numbers of T-cells in the lesions of injected *LDLR*$^{-/-}$ mice. Sections were stained with a T-cell specific anti-CD3 antibody, and values represent the numbers of CD3^{+} cells/ mm^{2} of total lesion area. All data are mean ± SEM values of all mice of each group.

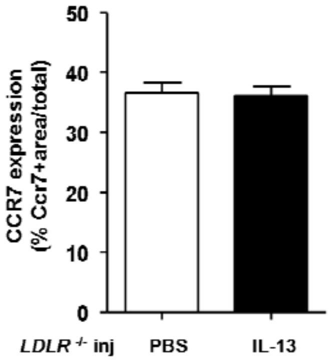

Fig S3. Equal expression of CCR7 in atherosclerotic lesions of injected *LDLR$^{-/-}$* mice. *LDLR$^{-/-}$* mice were fed an atherogenic diet for 16 weeks and received biweekly intraperitoneal injections with PBS (n=11) or IL-13 (n=13) during the last 5 weeks. CCR7 expression was assessed in lesions of injected *LDLR$^{-/-}$* mice by immunohistochemistry. Values represent the percentages of CCR7^{+} area/total lesion area. All data are mean ± SEM values of all mice of each group.

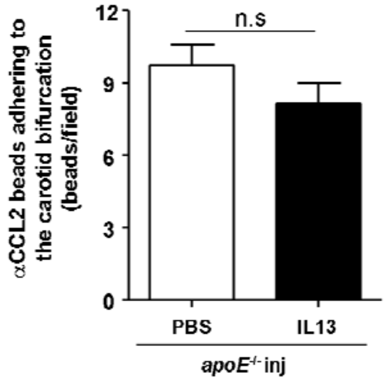

Fig S4. Equal expression of CCL2 by endothelial cells of injected *ApoE*$^{-/-}$ mice. *ApoE*$^{-/-}$ mice were fed an atherogenic diet for 6 weeks and received biweekly intraperitoneal injections with PBS (n=8) or IL-13 (n=8) during the last 2 weeks. At time of sacrifice, fluorescent anti-CCL2 beads were injected intravenously and the number of beads adhering to the carotid bifurcation was assessed by intravital microscopy. Values represent the numbers of beads/optical field. All data are mean ± SEM values of all mice of each group.

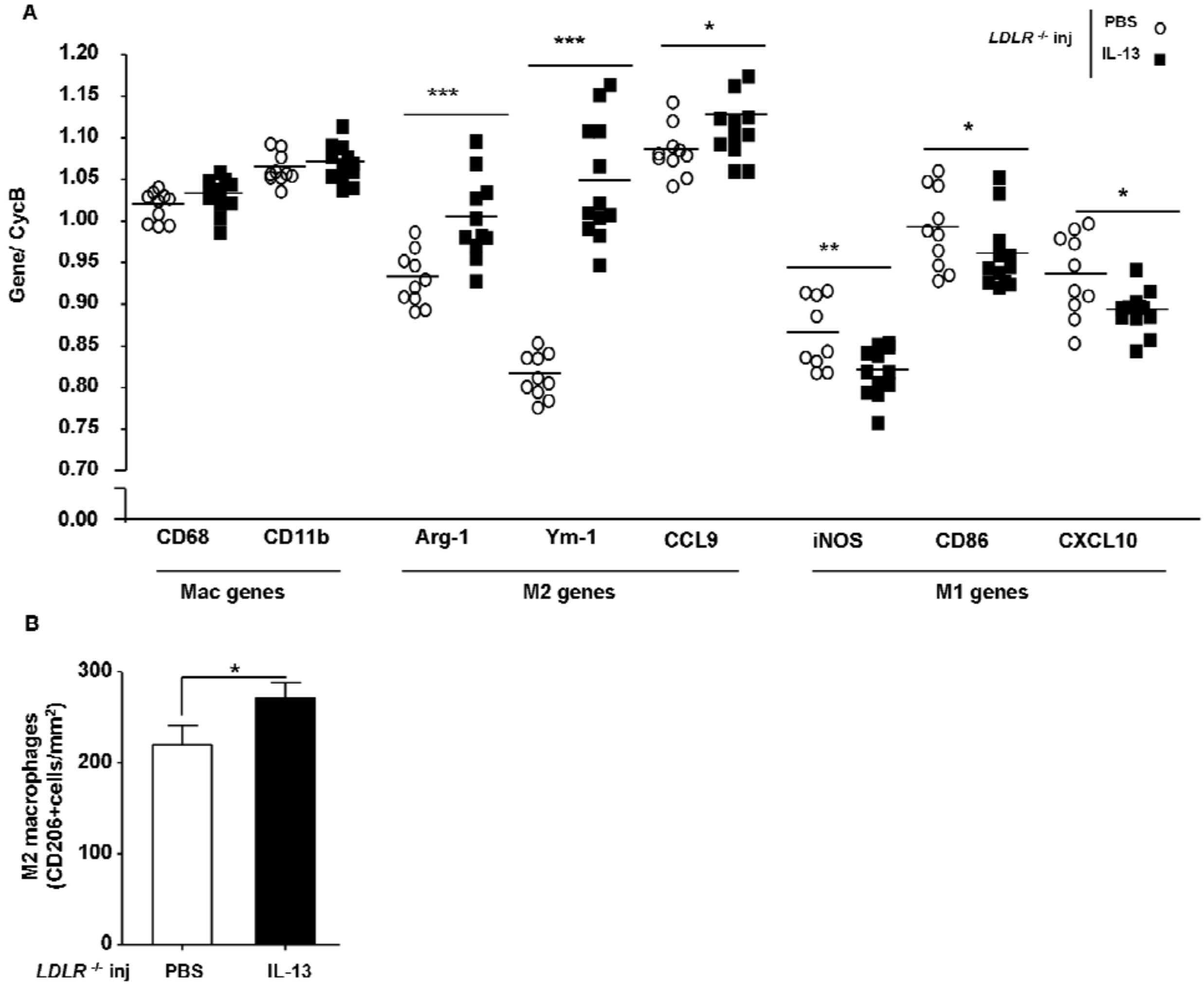

Fig S5. IL-13 administration induces alternatively activated M2 macrophages *in vivo*. *LDLR*$^{-/-}$ mice were fed an atherogenic diet for 16 weeks and received biweekly intraperitoneal injections with PBS (n=11) or IL-13 (n=13) during the last 5 weeks. **(A)** Increased expression of M2 genes in peritoneal exudate cells of *LDLR*$^{-/-}$ mice injected with IL-13. The expression of indicated genes was assessed in total peritoneal cells by quantitative RT-PCR. Data were normalized to CycB expression. Symbols indicate the normalized expression levels of individual mice, and horizontal bars indicate means of each group (*p=0.04, **p=0.0031, ***p=0.0001). **(B)** Increased M2 macrophages in lesions of *LDLR*$^{-/-}$ mice injected with IL-13. Sections were stained with an antibody against CD206, which is specifically expressed by M2 macrophages. Values represent the numbers of CD206^{+} cells/mm^2 of total lesion area (*p=0.036). All data are mean ± SEM values of all mice of each group.

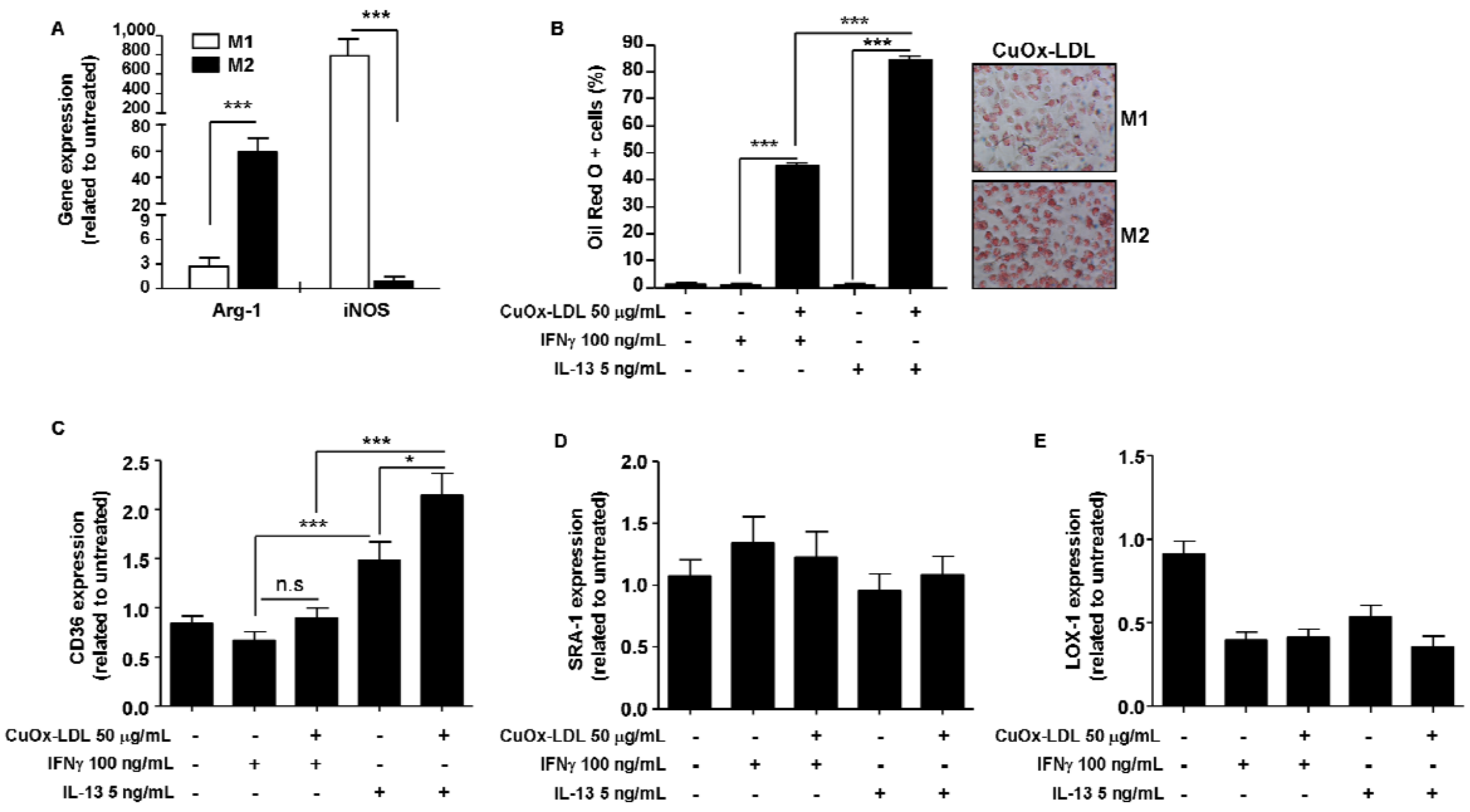

Fig S6. Alternatively activated macrophages (M2) exhibit increased uptake of CuOx-LDL *in vitro*. Thioglycollate-elicited macrophages were stimulated with IFN-γ or IL-13 into classically (M1) or alternatively (M2) activated macrophages, respectively. **(A)** The expression of Arginase-1 (Arg-1) and iNOS was analyzed by quantitative RT-PCR. Data were normalized to CycB expression and values represent fold increased expression over untreated cells. Data are mean ± SEM of two independent experiments performed in triplicates (***p=0.0001). **(B-E)** M1 and M2 macrophages were incubated with CuOx-LDL to generate foam cells. **(B)** Increased lipid uptake in M2-derived foam cells**.** Foam cells were stained with Oil Red O (ORO) and the percentages of ORO^+ cells/total cells were quantified. All data are mean ± SEM values of two independent experiments performed in quadruplicates (***p=0.0001). The expression of **(C)** CD36, **(D)** SRA-1 and **(E)** LOX-1 was analyzed by quantitative RT-PCR. Data were normalized to CycB expression and values represent fold increased expression over untreated cells. Data are mean ± SEM of three independent experiments performed in triplicates (*p=0.038, ***p=0.0001).

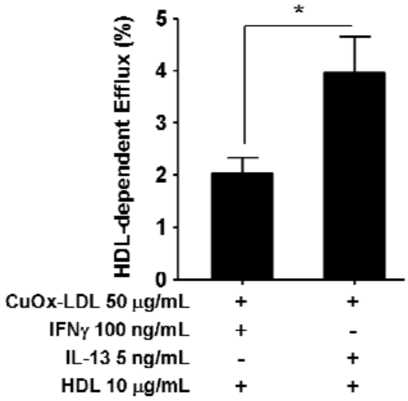

Fig S7. Increased HDL-dependent cholesterol efflux by foam cells stimulated with IL-13. Thioglycollate-elicited macrophages were incubated with CuOx-LDL plus 1 µCi of [^{3}H]-cholesterol for 24 hours and then stimulated with IFN-γ or IL-13 into classically (M1) or alternatively (M2) activated macrophage/foam cells, respectively. Subsequently, HDL-dependent efflux was assayed as described in Methods. Data represent percentages of HDL-dependent efflux/total efflux. Data are mean ± SEM of two independent experiments performed in quadruplicates (Student t-test *p=0.0178).

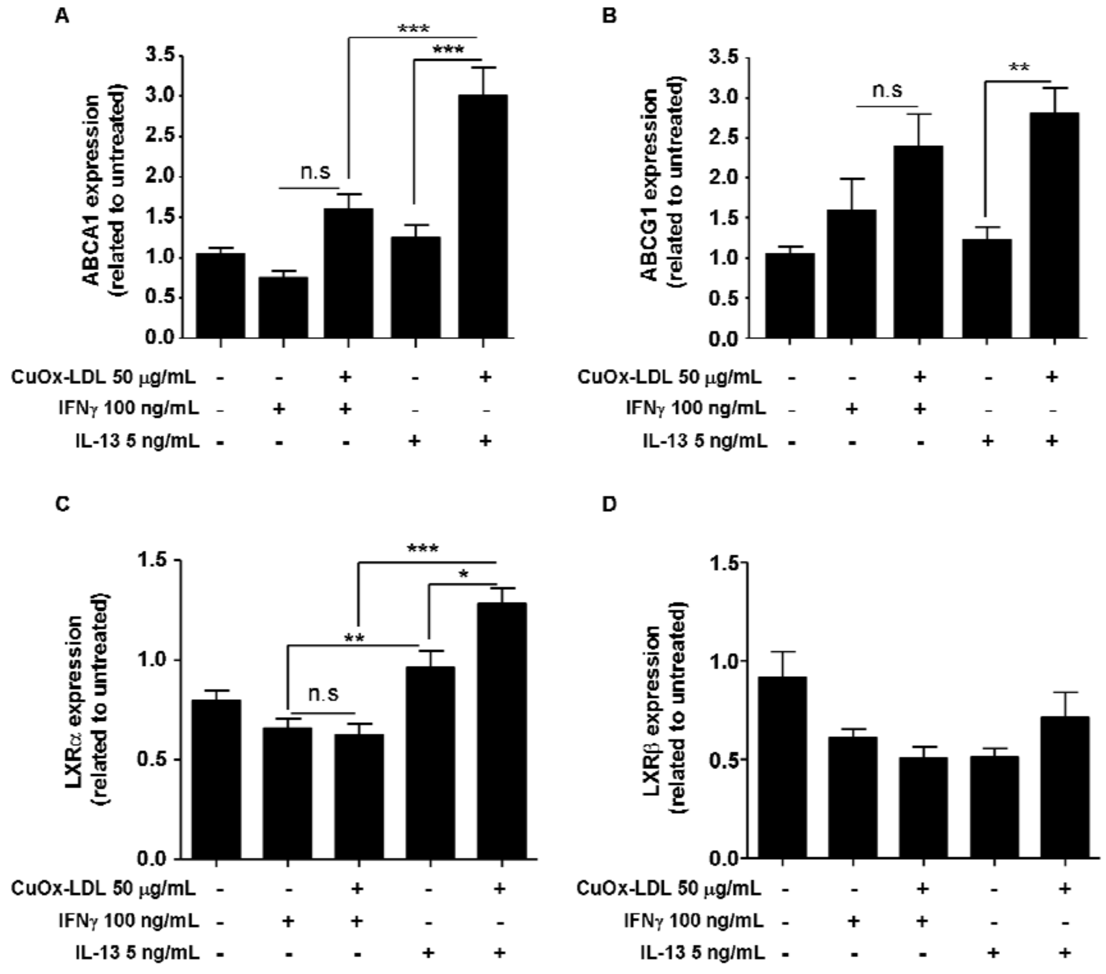

Fig S8. Gene expression of alternatively activated M2 macrophages *in vitro*. Thioglycollate-elicited macrophages were stimulated with IFN-γ or IL-13 into classically (M1) or alternatively (M2) activated macrophages, respectively, and then incubated with CuOx-LDL for 24h to generate foam cells. The expression of **(A)** ABCA1, **(B)** ABCG1, **(C)** LXRα and **(D)** LXRβ was analyzed by quantitative RT-PCR. Data were normalized to CycB expression and values represent fold increased expression over untreated cells. Data are mean ± SEM of three independent experiments performed in triplicates (*p=0.04, **p=0.003, ***p=0.0001).

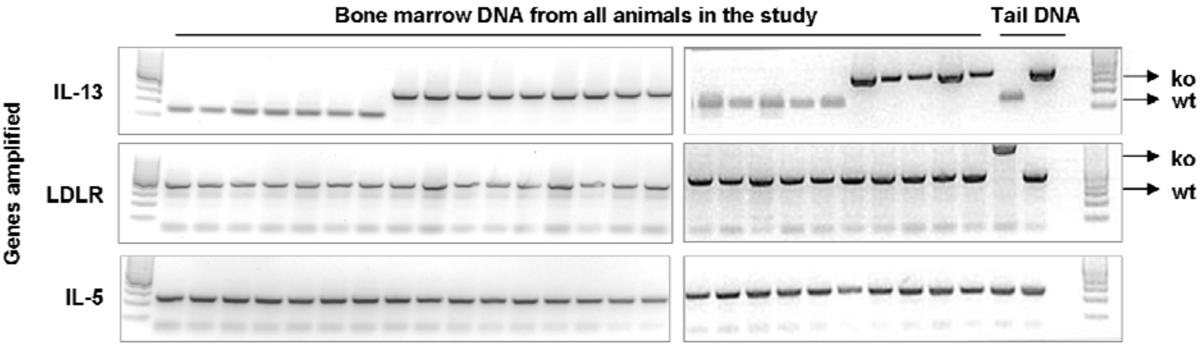

Fig S9. Successful bone marrow reconstitution in IL-13 chimeric mice. *LDLR*$^{-/-}$ mice were reconstituted with bone marrow cells isolated from either *IL-13*$^{+/+}$mice (n=12) or *IL-13*$^{-/-}$ mice (n=14) and fed an atherogenic diet for 16 weeks. At time of sacrifice bone marrow cells were collected from all mice and genomic DNA extracted and amplified for IL-13, LDLR and IL-5 genes. Tail DNA from a *IL-13*$^{+/+}$, *IL-13*$^{-/-}$ and LDLR$^{-/-}$ mouse were used as positive and negative controls.

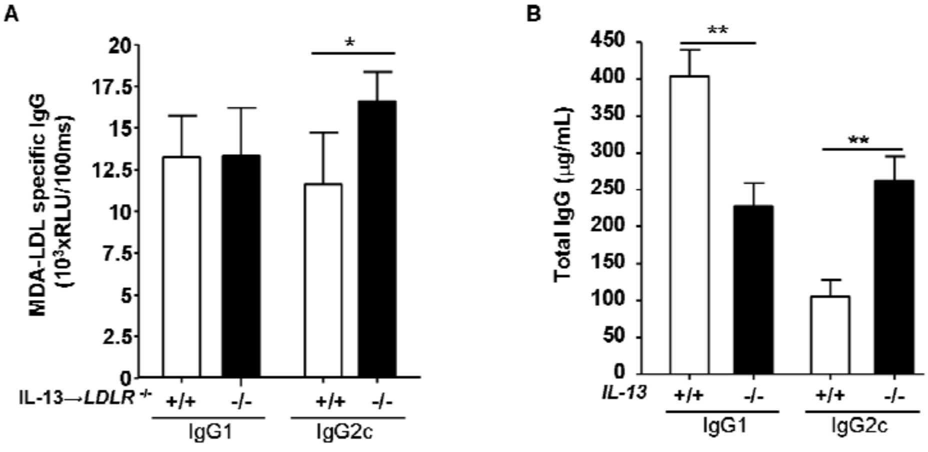

Fig S10. Effect of *IL-13*-deficiency on antibody isotype levels. (A) *LDLR*$^{-/-}$ mice were reconstituted with bone marrow from either *IL-13*$^{+/+}$ mice (n=12) or *IL-13*$^{-/-}$ mice (n=14) and fed an atherogenic diet for 16 weeks. At time of sacrifice blood was collected from all mice, and IgG1 and IgG2c antibodies against MDA-LDL were determined. Values represent relative light units (RLU)/100 ms (*p=0.04). **(B)** IL-13$^{-/-}$ mice have increased levels of total IgG2c and decreased levels of total IgG1 antibodies in serum. IgG isotypes in sera of IL-13$^{+/+}$ (n=8) or IL-13$^{-/-}$ mice (n=8) were quantified by ELISA. Data are presented as μg/mL of indicated IgG isotypes (**p=0.005). All values are mean ± SEM of all mice of each group.

SUPPLEMENTAL TABLES

Table SI
Overview of experimental data of the bone marrow transplantation study.

	IL-13$^{+/+}$ → *LDLR*$^{-/-}$ (n=12)	*IL-13*$^{-/-}$ → *LDLR*$^{-/-}$ (n=14)
**Atherosclerosis**		
Aortic origin (10^4 μm^2/sect.)	11.44 ± 2.03	21.86 ± 2.38**
En face (% of aorta)	4.72 ± 0.78	7.00 ± 1.27
**Metabolic parameters**		
Weight (g)	29.4 ± 0.78	29.2 ± 0.47
TC (mg/dL)	788.2 ± 85.3	859.8 ± 46.9
TG (mg/dL)	591.2 ± 79.6	606.1 ± 57.4
**Serum antibody titers**		
Total IgM (mg/mL)	1.64 ± 0.15	1.64 ± 0.11
Total IgG1 (mg/mL)	2.37 ± 0.55	2.00 ± 0.50
Total IgG2c (mg/mL)	1.01 ± 0.14	1.55 ± 0.22*
**Splenic cytokines**[A]		
IL-4 (ng/mL)	12.44 ± 2.33	2.20 ± 0.53***
IL-5 (ng/mL)	0.75 ± 0.03	0.91 ± 0.14
IL-10 (ng/mL)	6.66 ± 0.62	2.92 ± 0.35***
IL-13 (ng/mL)	33.68 ± 3.34	2.23 ± 0.12***
IFN-γ (ng/mL)	142.6 ± 4.55	135.0 ± 5.10
**Characterization of splenocytes** [B]		
Total Spleen cells (x10^6)	8.99 ± 0.56	9.89 ± 0.63
CD43$^+$ T-cells (% of total)	22.05 ± 0.51	22.46 ± 0.55
CD220$^+$ B-cells (% of total)	58.14 ± 1.84	55.66 ± 1.61
CD43'IgM' B1-cells (% of B-cells)	13.53 ± 1.18	14.70 ± 0.97
CD220$^+$IgM$^+$ B2-cells (% of B-cells)	84.47 ± 1.21	83.20 ± 0.93

Atherosclerosis in the aortic origin was analyzed by cross-sections through the aortic origin and values represent the average μm^2/section. *En face* measurements are given in percent lesion area of the aorta. TC, total serum cholesterol; TG, serum triglycerides. [A]Cytokine secretion in supernatants of splenocyte cultures following stimulation with anti-CD3/CD28. Cellular populations of [B]Splenocytes were analyzed by flow cytometry Values that are statistically different from the control group. (*p=0.029; **p=0.0023; ***p=0.0001). Data are mean ± SEM.

Table SII
Primer sequences

Gene	Forward	Reverse
CycB	CAGCAAGTTCCATCGTGTCATCAAGG	GGAAGCGCTCACCATAGATGCTC
CD11b	CAAGTGCCTGTCACACTGAGC	TGCAACAGAGCAGTTCAGCAC
CD68	TGGCGCAGAATTCATCTCTTC	GGTCAAGGTGAACAGCTGGAG
Arg-1	GTGAAGAACCCACGGTCTGT	CTGGTTGTCAGGGGAGTGTT
Ym-1	GGGCATACCTTTATCCTGAG	CCACTGAAGTCATCCATGTC
CCL9	TACTGCCCTCTCCTTCCTCA	TTGAAAGCCCATGTGAAACA
iNOS	AATCTTGGAGCGAGTTGTGG	CAGGAAGTAGGTGAGGGCTTG
CD86	CACGAGCTTTGACAGGAACA	TTAGGTTTCGGGTGACCTTG
CXCL10	GGTCTGAGTGGGACTCAAGG	GTGGCAATGATCTCAACACG
ABCA-1	GAGCAAAGCCAAGCATCTTC	AGCAGGGACCACATAATTGC
ABCG-1	TTCCGGAAGGTCTCCTGCTAC	ATCATGGCCTCCTGAACAGTG
LOX-1	GATGTTAGCCCAGCAGAAGG	GTCCAGCTTCCGGGTGAT
LXRα	GGATAGGGTTGGAGTCAGCA	GCTCAGCACGTTGTAATGGA
LXRβ	CTTCGGGCTTCCACTACAAC	CTTCCGAATCTGCTCCTCAG
CD36	GGAGCAACTGGTGGATGGTT	TTGAGACTCTGAAAGGATCAGCA
SRA-1	GAACAAGCGCACGTGGAAC	CCTTCAGTCTGAGGTCGTTGG
